# Fluorescence Lifetime Readouts of Troponin-C-Based Calcium FRET Sensors: A Quantitative Comparison of CFP and mTFP1 as Donor Fluorophores

**DOI:** 10.1371/journal.pone.0049200

**Published:** 2012-11-09

**Authors:** Romain Laine, Daniel W. Stuckey, Hugh Manning, Sean C. Warren, Gordon Kennedy, David Carling, Chris Dunsby, Alessandro Sardini, Paul M. W. French

**Affiliations:** 1 Institute of Chemical Biology (ICB), Imperial College of Science, Technology & Medicine, Institute of Chemical Biology (ICB), London, England; 2 Photonics Group, Blackett Lab, Imperial College of Science, Technology & Medicine, London, England; 3 Medical Research Council (MRC) Clinical Sciences Centre, Imperial College of Science, Technology & Medicine, London, England; University of Oldenburg, Germany

## Abstract

We have compared the performance of two Troponin-C-based calcium FRET sensors using fluorescence lifetime read-outs. The first sensor, TN-L15, consists of a Troponin-C fragment inserted between CFP and Citrine while the second sensor, called mTFP-TnC-Cit, was realized by replacing CFP in TN-L15 with monomeric Teal Fluorescent Protein (mTFP1). Using cytosol preparations of transiently transfected mammalian cells, we have measured the fluorescence decay profiles of these sensors at controlled concentrations of calcium using time-correlated single photon counting. These data were fitted to discrete exponential decay models using global analysis to determine the FRET efficiency, fraction of donor molecules undergoing FRET and calcium affinity of these sensors. We have also studied the decay profiles of the donor fluorescent proteins alone and determined the sensitivity of the donor lifetime to temperature and emission wavelength. Live-cell fluorescence lifetime imaging (FLIM) of HEK293T cells expressing each of these sensors was also undertaken. We confirmed that donor fluorescence of mTFP-TnC-Cit fits well to a two-component decay model, while the TN-L15 lifetime data was best fitted to a constrained four-component model, which was supported by phasor analysis of the measured lifetime data. If the constrained global fitting is employed, the TN-L15 sensor can provide a larger dynamic range of lifetime readout than the mTFP-TnC-Cit sensor but the CFP donor is significantly more sensitive to changes in temperature and emission wavelength compared to mTFP and, while the mTFP-TnC-Cit solution phase data broadly agreed with measurements in live cells, this was not the case for the TN-L15 sensor. Our titration experiment also indicates that a similar precision in determination of calcium concentration can be achieved with both FRET biosensors when fitting a single exponential donor fluorescence decay model to the fluorescence decay profiles. We therefore suggest that mTFP-based probes are more suitable for FLIM experiments than CFP-based probes.

## Introduction

Calcium is a ubiquitous intracellular secondary messenger involved in several fundamental cellular processes [1], [2], [3] such as secretion, proliferation and membrane excitability. It is essential for the cell to regulate the very low intracellular calcium level (in the order of 100 nM) and control its level when Ca^2+^-dependent pathways are activated, during which it rises to roughly 1 µM [2]. In order to monitor and visualize intracellular calcium concentrations, genetically encoded calcium indicators (GECIs) [4] offer some key advantages in comparison to synthetic calcium dyes. In particular GECIs do not need to be loaded into the cell, they are directly expressed by the transfected cells. They can also be targeted to specific sub-cellular organelles and do not leak out of the cells, allowing long-time-course recording. Two structurally different classes of GECI can be recognized: a Förster resonance energy transfer (FRET)-based type that relies on the use of calcium binding element interposed between two fluorescent proteins (FP) and a second class where the calcium sensor uses a single FP.

The latter class comprises sensors such as G-CaMP [5] and Pericam [6] which respond to calcium binding by changing their fluorescence intensity. In an effort to improve and expand the hue range of G-CaMP type sensors, which are based on circular permutated GFP, a recent study has published a colony-based screen for Ca^2+^-dependent fluorescent changes [7]. This screen has produced a new set of sensors, called GECO, with different calcium dynamic range and fluorescent hues. CatchER [8] has been developed for detecting high calcium in the endoplasmic reticulum and although it is based on a single FP, it differs from the other member of this class of sensors in the fact that the calcium binding site has been introduced into the eGFP itself, adjacent to the chromophore.

The most widely employed Calcium FRET sensors are the Cameleon sensors [9]. They comprise a fusion of the calmodulin protein and the calmodulin-binding domain of myosin light chain kinase M13 inserted between two fluorescent proteins such as CFP and YFP. Upon binding of calcium to calmodulin, the M13 chain binds to the calmodulin protein, bringing the two fluorophores into close proximity and allowing energy transfer to occur. However calmodulin is a ubiquitous signalling protein and may interfere with the expressed Cameleon sensors and at the same time the over-expressed sensors may also deregulate cell signalling [10]. In order to bypass this issue, a different set of calcium FRET biosensors that employ Troponin C as the calcium-binding moiety have been generated [11], [12]. Troponin C is selectively expressed in skeletal muscle cells and therefore does not interfere with normal cellular processes when introduced in cell lines not derived from myocytes. In particular the TN-L15 sensor developed by Heim et al. [11] consists of a chicken skeletal muscle Troponin C segment inserted between the fluorescent proteins ΔC11CFP (CFP truncated at its C-terminus by 11 amino acids) and the improved yellow mutant of YFP called Citrine [13]. 14 amino-acids of the N-terminus of the Troponin C fragment were removed in order to optimize the efficiency of the energy transfer. The binding of calcium to the binding sites (apparent dissociation constant of 1.2 µM, [11]) induces a conformational change bringing the two fluorophores closer together, and allowing energy transfer to occur.

The efficiency of the Förster resonance energy transfer depends on the relative geometry between the two fluorophores (distance and orientation) as well as their spectral properties. The theory developed by Förster gives expressions for the FRET efficiency and introduces the so-called “Förster distance” [14]. Equation (1) highlights the dependence of the FRET efficiency (*E_FRET_*) on the distance separating the two fluorophores (*r*) and the Förster distance (*R_0_*).
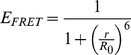
(1)The Förster distance (*R_0_*) is characteristic of the pair of fluorophores used as donor/acceptor and their relative orientation as shown in equation (2), where *κ^2^* is the orientation factor, *J(λ)* represents the spectral overlap between the donor emission spectrum and the acceptor absorption spectrum, *η* is the donor quantum yield and *n* is the refractive index of the medium.
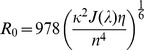
(2)The orientation factor describes the angular dependence of the energy transfer. It is maximal when the dipoles of the fluorophores are collinear and becomes zero when they are orthogonal. As the Förster distance is usually in the order of 5–10 nm, nanometre-scale conformational changes in the probe can be read out through changes in the FRET efficiency.

The methods most widely applied to read-out FRET efficiency are intensity-based (spectral ratiometric) [11], [15], [16] or fluorescence lifetime-based measurements [17], [18], [19]. Spectral ratiometric read-outs are sensitive to spectral cross-talk, requiring additional calibration samples for quantitative measurements [14], [20], and can be more severely impacted by photobleaching, optical scattering and the inner filter effect, which can be important when measuring signals in biological tissue [21]. We therefore believe it is useful to characterise the performance of FRET biosensors using the fluorescence lifetime approach, which only requires measurements of the donor emission.

In fluorescence lifetime-based FRET measurements the energy transfer can be observed by a decrease of the lifetime of the donor fluorophore. In the case of a sensor molecule with discrete “open” (no FRET) and “closed” (FRET) conformations, a monoexponential donor fluorophore should generally present a bi-exponential fluorescence decay profile. Under these assumptions the contribution of the short lifetime component to the observed decay profile depends on the fraction of donor fluorophores undergoing FRET and its lifetime is related to the FRET efficiency, *E_FRET_*, by equation (3), where *τ_DA_* and *τ_D_* are the lifetime of the donor fluorophore in presence and in absence of the acceptor respectively.
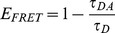
(3)More generally, the sensor may still undergo residual FRET in its “open” conformation. In this case we describe the “open” form of the sensor as the “low FRET” conformation and the “closed” form as the “high FRET” conformation.

Currently the most widely used pair of genetically encoded fluorophores for FRET experiment is the cyan/yellow fluorescent proteins. The cyan fluorescent protein (CFP) family has expanded significantly since the first variant [22], today notably including the more stable ECFP [9], brighter Cerulean [23], or the reef coral-derived AmCyan [24]. The complex fluorescence decay of most CFPs [25], [26], however, complicates the quantitative interpretation of fluorescence lifetime measurements when it is employed as a FRET donor. To address this issue, mono-exponential cyan variants, such as mTurquoise [27] or the *Clavularia* coral-derived monomeric teal fluorescent protein (mTFP1 [28]) were created. We have followed previous work and created a new calcium FRET probe by substituting CFP in TN-L15 with mTFP1, which approximates well to a monoexponential fluorescence decay model and offers a better spectral overlap with the yellow fluorescent protein [29], [30], being slightly red-shifted compared to the cyan variants. We note that Geiger et al. [31] have previously suggested that a Troponin C-based calcium FRET sensor would benefit from a donor presenting a mono-exponential fluorescence decay profile for fluorescence lifetime readouts. However they did not demonstrate this.

To better understand the utility of this new calcium FRET biosensor, called mTFP-TnC-Cit, we have undertaken a protocol that we developed for preparing cytosol preparations from mammalian cells [32] that provides a convenient means to produce bulk solutions of the calcium FRET biosensors. These cytosol preparations provide a biologically relevant system for studying proteins in aqueous phase and can be made more readily and rapidly than bulk solutions based on protein purification from bacterial preparations. The robust data afforded by fluorescence lifetime measurements from solution phase can provide quantitative information on the fraction of molecules undergoing FRET and the distance separating the donor and the acceptor [33] whilst only requiring one additional measurement of the donor fluorescence decay profile in the absence of acceptor. By carrying out a fluorescence lifetime-resolved calcium titration of both FRET biosensors, we have been able to explore the precision of their readouts and measure their dissociation constants.

## Materials and Methods

### Plasmids and preparation of mTFP-TnC-Cit and ΔC11CFP

eGFP (enhanced green fluorescent protein) was expressed from pEGFP-C1 (Clontech). The TN-L15 sensor, cloned into the mammalian expression vector pcDNA3, was a generous gift from O. Griesbeck [11]. mTFP1 was obtained from Allele Biotechnology (pmTFP1-N vector). mTFP-TnC-Cit was constructed by substituting a single fluorophore from the original TN-L15 sensor. Since TN-L15 is inserted between *Bam*H1 and *Eco*R1, a double digestion of the TN-L15 vector with *Bam*H1/*Eco*R1 enabled us to isolate the TN-L15 insert and the pcDNA3 backbone. The insert was then digested with *Sph*1 to separate the TnC–Cit fragment from the CFP fragment. mTFP1 was amplified by PCR (see Figure S1 for the sequences of forward and reverse primers). It was then digested with *Bam*H1 and *Sph*1. The reverse primer was designed such that it includes the *Sph*1 restriction site and the 10 base pairs preceding the STOP codon. The STOP codon was deleted to allow read-through of the entire sensor, and a guanine was introduced in order to keep the open reading frame in-frame with that of the Troponin C – Citrine fragment. The mTFP1 fragment, the Troponin C – Citrine fragment and the backbone of pcDNA3 were ligated. At each step of the cloning of mTFP-TnC-Cit the DNA was run on a 1% agarose gel. DNA bands were purified using a gel purification kit (Qiagen).

XL10 Ultracompetent cells were transformed with the new sensor by heat shock procedure. DNA was extracted and purified (QIAprep Spin Miniprep Kit, Qiagen) and diagnostically digested with ECO0109I and *Eco*R1. The ECO0109I restriction site is present within the open reading frame of mTFP-TnC-Cit and therefore reveals the presence of clones correctly expressing mTFP-TnC-Cit. A second diagnostic digest with *Bam*H1/*Eco*R1 was carried out in order to confirm the presence of the whole insert. DNA was then amplified, purified (Plasmid Maxi Kit, Qiagen) and verified by sequencing.

ΔC11CFP was prepared using the TN-L15 plasmid as a template in a PCR reaction (see supporting material S2 for primer sequences). The reverse primer introduced a STOP codon at the end of the CFP open reading frame followed by an *Eco*R1 restriction site. The digested fragment was then re-introduced into pcDNA3.

### Cytosol preparations

Plasmids coding for the calcium FRET sensors or single fluorescent proteins were transiently transfected into SV40-transformed human embryonic kidney cells (HEK293T) cells using polyethylenimine (PEI), as previously described [32]. Briefly, sub-confluent HEK293T cells cultured in 175 cm^2^ flask were transfected with 50 µg of DNA (2.5 µL of PEI per 1 µg of DNA in Opti-MEM®, Invitrogen) and incubated for 8–10 hours at 37°C. 24–48 hours post-transfection, cells were detached and homogenized (Ultra-turrax) in lysis buffer (50 mM TrisCl, pH = 7.4, 50 mM mannitol, 40 µM of EDTA and protease inhibitors cocktail, *cOmplete EDTA-free Cocktail*, Roche). Nuclei and debris were spun down at 500 g and the supernatant stratified on membrane re-suspension buffer (50 mM TrisCl, pH = 7.4, 300 mM mannitol and protease inhibitors cocktail, *cOmplete EDTA-free Cocktail*, Roche) before ultracentrifugation at 40,000 rpm for 45 minutes. The cytosol preparation was then recovered from the later spin and then concentrated by centrifugation at 3,000 rpm with a spin column (30 kDa cut off filter for FRET sensors and 10 kDa for single fluorophores, Vivaspin 6 from Sartorius Vivascience).

### Multidimensional fluorometer

All fluorescence measurements were undertaken using a home-built multidimensional fluorometer resolving fluorescence lifetime, spectrum and polarization [34]. The detection system combines Time-Correlated Single Photon Counting (TCSPC, Becker & Hickl, SPC 730) for precise lifetime measurements with a scanning monochromator and automated polarizers for spectrally and polarization-resolved fluorescence emission analysis. The excitation source is a picosecond fibre laser-pumped supercontinuum (SPC400-2PP, Fianium) source that is spectrally tuneable to provide the optimum excitation wavelength. The instrument response function (IRF) is acquired at the excitation wavelength using a colloidal silica suspension (LUDOX® SM-30, Aldrich). All fluorescence lifetime measurements were carried out by exciting the sample with a vertical polarization and measuring its emission at the magic angle polarization. The temperature was controlled by using a cuvette holder connected to a temperature-controlled water bath (Grant, LTD6G & LTD6/20).

### Fluorescence lifetime measurements of single fluorophores

Purified eGFP (rEGFP protein, BD Clontech) was diluted in membrane re-suspension buffer (TrisCl, pH = 7.4) to 10 µM. Both eGFP cytosol preparation and purified eGFP were excited at 490 nm (10 nm bandwidth) and measured at 510 nm (10 nm bandwidth), at room temperature.

The samples of ΔC11CFP and mTFP1 were prepared by diluting their respective cytosol preparations in the Component A buffer of the Calcium calibration kit #1 (Invitrogen) in a ratio 1∶10. The acquisition was performed at 37°C. The ΔC11CFP sample was excited at 430 nm (10 nm bandwidth) and detected at 475 nm (10 nm bandwidth). mTFP1 was excited at 460 nm (10 nm bandwidth) and detected at 490 nm (10 nm bandwidth).

### Measurements of the temperature dependence of the fluorescence lifetimes

The temperature dependence of the fluorescence lifetime for both ΔC11CFP and mTFP1 was studied in the multidimensional fluorometer by stabilising the sample at temperatures ranging from 20°C to 50°C in 5°C steps. The sample was prepared by diluting the cytosol preparation 1∶10 in the Component A buffer of the Calcium calibration kit #1 from Invitrogen. The error on the temperature is of the order of ±1°C. ΔC11CFP was excited at 430 nm (10 nm bandwidth) and its fluorescence emission was collected at 475 nm (10 nm bandwidth). mTFP1 was excited at 460 nm (10 nm bandwidth) and measured at 490 nm (10 nm bandwidth). All measurements were carried out by adjusting the integration time such that the number of photons in the maximum of every fluorescence decay profile reached 10,000.

### Fluorescence lifetime versus emission wavelength

The dependence of the fluorescence lifetime with the emission wavelength of ΔC11CFP and mTFP1 was carried out on cytosol preparations (diluted 1∶10 in the Component A buffer of the Calcium calibration kit #1 from Invitrogen) using the multidimensional fluorometer. All measurements were carried out at 37°C and the integration time was adjusted such that the number of photons in the maximum of each fluorescence decay profile reached 10,000. ΔC11CFP and mTFP1 were excited at 430 nm (10 nm bandwidth) and 460 nm (10 nm bandwidth) respectively.

### Calcium titration

The titration was achieved by fixing the concentration of the free calcium available for the sensor by buffering of the total calcium with calcium chelator (EGTA) [35]. Given that the binding affinity of EGTA is temperature dependant, the temperature of the sample had to be strictly controlled and we chose to work at 37°C. Calcium titration was carried out with the Calcium Calibration kit #1 (Invitrogen) in the absence of Magnesium at 37°C, pH = 7.2 (MOPS). Free calcium levels were calculated using the complete mathematical description of the constant of affinity model as shown in equation (4).
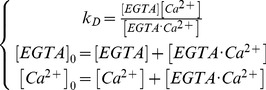
(4)Where *k_D_* is the dissociation constant, [*Ca^2+^*], [*EGTA*] and [*EGTA·Ca^2+^*] are respectively the concentrations of free calcium ion, calcium-free EGTA and calcium-bound EGTA. [*EGTA*]_0_ and [*Ca^2+^*]_0_ are respectively the total concentration of EGTA and calcium disregarding their status of binding.

A value of 107.9 nM was used for the binding affinity of EGTA (*k_D_*) at 37°C, as previously reported [35]. The concentrations of the calcium free in solution ([*Ca^2+^*] in µM) used for the titration were 0, 0.02, 0.05, 0.1, 0.2, 0.5, 1, 2, 5, 10, 20 and 40 µM. Samples were diluted 1∶10 in the appropriate buffers.

The single fluorophore sample (ΔC11CFP and mTFP1) measurements were prepared by diluting the cytosol preparation 1∶10 in the Component A buffer of the Calcium calibration kit #1 (Invitrogen).

### Data analysis, global analysis and fitted models

Data analysis was performed with discrete exponential models (see equation (5)), using the TRFA Data processor Advanced 1.4 (SSTC) software package.
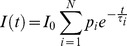
(5)In equation (5), *τ_i_* and *p_i_* represent the lifetime and the initial fractional contribution of the *i*-th exponential component of the decay respectively. *p_i_* is proportional to the number of fluorophores with lifetime *τ_i_*.

The fluorescence decay analysis used the method of non-linear minimization of the reduced *χ^2^* parameter (defined by equation (6)).
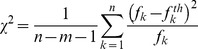
(6)In equation (6), *f_k_* and *f^th^_k_* are the values of the measured and theoretical (from the model) fluorescence signal in time bin *k* respectively. *n* is the total number of time bins analysed and *m* is the number of fitted parameters. Here this criterion is used to describe the quality of the fit.

Average fluorescence lifetimes were calculated using equation (7), i.e. the photon-weighted average lifetime, as described by Lakowicz [14].
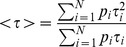
(7)The relative contribution of a particular decay component to the total fluorescence signal (*a_k_*) is calculated using equation (8). It takes into account the contribution in population (*p_k_*) as well as its fluorescence lifetime (*τ_k_*).
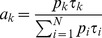
(8)For the calcium titration, a two population model was adopted: one “high FRET” (or “closed”) conformation and one “low FRET” (or “open”) conformation. Fluorescence decay data corresponding to each state were fitted using a model with the same number of exponential components as that of the donor only. For TN-L15 (donor CFP), a 4 exponential model was adopted (2 components for each conformation). A bi-exponential model was chosen for mTFP-TnC-Cit. The number of decay components was selected based on an analysis of the fluorescence from the donor alone, as presented in the results section. The fluorescence lifetime components were fitted globally across the whole titration dataset (e.g. all concentrations) and only the contributions of these components were allowed to vary at each concentration. For the model chosen to fit the TN-L15 titration (4-component model), 4 lifetime components and 48 contribution factors (4 contribution factors at each of 12 concentrations) were used in the global fitting. For mTFP-TnC-Cit the global model comprises 2 lifetime components and 24 contribution factors (2 pre-exponential factors at each of 12 concentrations).

Uncertainties on the presented fluorescence decay parameters were calculated in one of two ways. The first used the confidence intervals calculated by the TRFA fitting software. It provides an analysis of the 67% confidence interval using an exhaustive search method. The second was based on repeated acquisition of decay data from the same sample (3 replicates) that were fitted separately.

The global analysis with the constant ratios uses the model described in equation (9). The ratios *r_21_* and *r_43_* are globally fitted across the dataset.
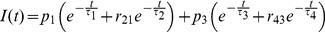
(9)This model describes a bi-exponential donor fluorescence decay in two different conformation (“low FRET” and “high FRET”). Furthermore we assume that the relative contributions of the two lifetime components within the same conformation are the same, resulting in *r_21_ = r_43_*. This is achieved by linking the values of the two ratios within the fitting model. In this case and if the components 1 and 2 represent the population in the “high FRET” conformation then the total fraction of “closed” conformation *q_1_* can be written as equation (10).
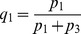
(10)This model therefore results in 5 parameters being fitted globally (*r_21_* (* = r_43_*), *τ_1_*, *τ_2_*, *τ_3_* and *τ_4_*) and 24 contribution factors (*p_1_* and *p_3_* at each one of the 12 calcium concentrations).

The phasor analysis was performed using a custom-written routine in MATLAB®, following previous work [36], [37] using the equations (11) and (12).
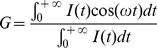
(11)

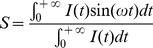
(12)The calibration of the phasor plot was achieved by using the scatterer dataset (reference lifetime of zero). The calibration was tested against a sample of Rhodamine 6G (Lambda Physik) in water (2 µM) leading to a modulation lifetime *τ_m_ = 4.011 ns* and phase lifetime *τ_φ_ = 4.012 ns* [14] which are in excellent agreement with each other and with the value obtained from the fitting in the time-domain using the TRFA software package (*4.011 ns*). The phasor point obtained from the Rhodamine 6G dataset therefore lies perfectly on the universal circle (data not shown).

The titration data were analysed by fitting a straight line through the phasor points [36]. For this purpose the gradient and Y-intercept of the line were analytically expressed as a function of the two lifetimes corresponding to the two intersects of this line with the universal circle. These two lifetimes were then obtained by the least square fitting method with a custom written MATLAB® routine. The errors on the lifetimes are given by the 67% confidence interval bounds. Relative contributions of each of the two lifetime components were then calculated for each data point. The errors on the fractions were obtained from the experimental repeats.

### Titration model

In order to estimate the dissociation constant and the position of the endpoints from the various experimental titration curves obtained, we fitted the data to the model described in equation (13), using the *cftool* function in MATLAB®.
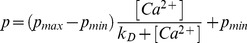
(13)Here *p* is the variable used to represent the fraction of sensor molecules in the “closed” conformation, [*Ca^2+^*] is the free calcium concentration, *k_D_* is the dissociation constant, *p_min_* is the value of *p* at [*Ca^2+^*]* = 0 µM* and *p*_max_ its value at [*Ca^2+^*]*>>k_D_*. The introduction of *p_min_* and *p_max_* in the model takes into account the fact that *p* may not vary from 0 to 1.

When fitting the experimental data to the model described by equation (13), it was necessary to define the relative weightings of the data points. For the data points at [*Ca^2+^*]* = 0 µM* and [*Ca^2+^*]* = 40 µM*, the standard deviation of the parameter *p* obtained from the experimental repeats was used. For all other concentrations, a linear interpolation between the measured standard deviation at the two endpoints was used.

When using a measured value of *p* to calculate [*Ca^2+^*] and to investigate the effect the error on *p* has on the calculated calcium concentration, we rearranged equation (13) to yield equation (14).
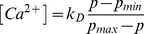
(14)


### Live-cell FLIM experiment

HEK293T (human embryonic kidney) cells were maintained at 37°C in a 5% CO_2_ vapour-saturated incubator in Dulbecco's modified Eagle medium (DMEM), 4.5 g/L glucose supplemented with 10% Foetal Bovine Serum (FBS), 2 mM glutamine and Pen/Strep until confluency. Cells were then plated on poly-L-lysine treated glass-bottom dish (35 mm diameter, from MatTek). After 24 hours they were transiently transfected with either TN-L15 or mTFP-TnC-Cit using the PEI protocol (1 µg of DNA and 2.5 µL of PEI per dish in Opti-MEM®, Invitrogen).

The live-cell experiments were carried out using a wide-field (Olympus IX81) microscope that was similar to that described previously [38]. The CFP in TN-L15 was excited at a wavelength of 435 nm and the CFP fluorescence was measured using a spectral filter centred at 482 nm (Semrock 482/35). For mTFP-TnC-Cit the mTFP1 was excited at 445 nm (Semrock 445/45) and the mTFP fluorescence was measured at 494 nm (Semrock 494/20).

Cells were imaged 48 hours after transfection in Calcium-free physiological solution (130 mM NaCl, 5 mM KCl, 0.5 mM MgCl_2_, 10 mM Hepes buffered at pH = 7.2, 1 g/L of glucose). The calcium calibration protocol has been previously described by Palmer et al. [39]. Briefly, a baseline level was acquired for 200 s (taking a FLIM image every 20 s), then EDTA and ionomycin were added to the imaging medium (10 mM EDTA and 5 µM ionomycin final concentrations) and the cells were imaged for 400 s (taking an image every 20 s). The cells were then washed and exposed to CaCl_2_ and ionomycin (10 mM CaCl_2_ and 5 µM ionomycin final concentration). The cells were finally imaged for another 400 s (taking an image every 20 s).

Data analysis was performed by an in-house MATLAB® routine using the method of non-linear minimization of the reduced *χ^2^* parameter. A measurement of DASPI (Radiant Dye laser) at 20 µM in water was used as a short lifetime (130 ps) reference for correction of the instrument temporal response function using reference re-convolution. Each frame of the time-course was fitted independently on a pixel-wise basis, after applying the appropriate intensity threshold. The average over all pixels in the cells was calculated leading to a single lifetime value for each frame of the time-courses. Then the averages over all frames of each time-course were calculated (baseline, EDTA, Ca^2+^). The mean and standard deviation of these 3 lifetimes were calculated over the repeats (N = 4).

## Results

### Validation of the cytosol preparation

**Figure 1:**
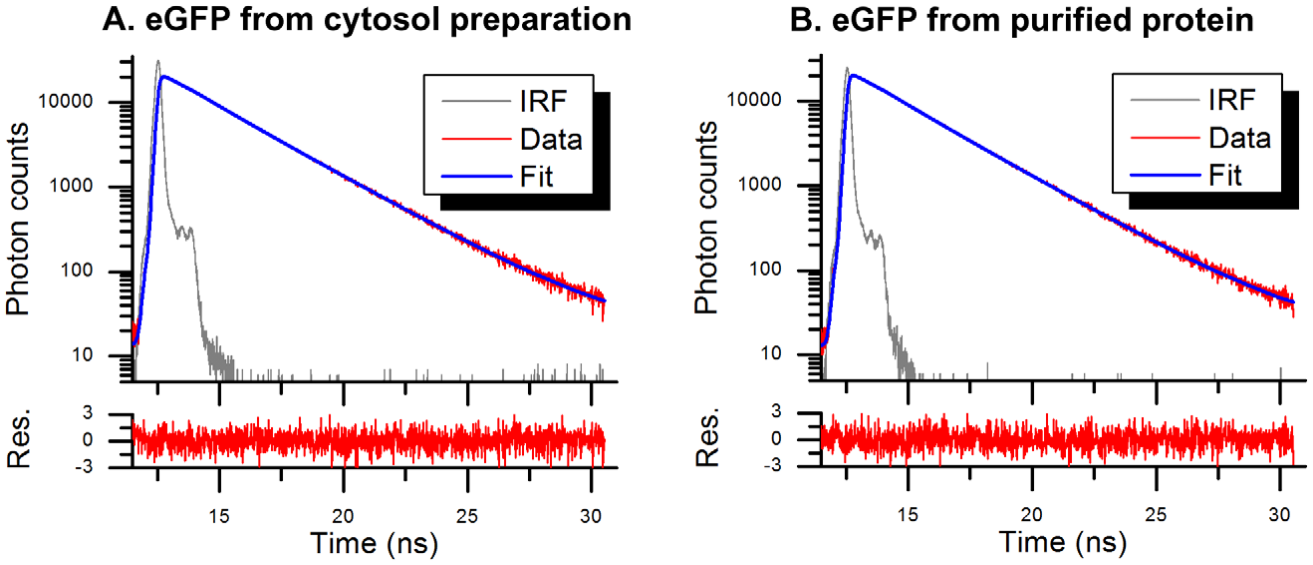
Fluorescence decays of eGFP at 21°C. (A) eGFP from cytosol preparation of HEK293T cells expressing eGFP. (B) purified eGFP. IRF: Instrument Response Function. Res. : residuals.

The use of cytosol preparation instead of purified protein for time-resolved fluorescence analysis was tested by comparing the fluorescence decays of eGFP protein in cytosol preparation with a purified sample. Figure 1 shows the fluorescence decay, fitting and residuals from the fitting of both samples. Table 1 shows the results of time-resolved fluorescence measurements.

**Table 1:** Results of time-resolved fluorescence analysis of eGFP obtained from cytosol preparation and purified eGFP.

	*p_1_*	*τ_1_ (ns)*	*p_2_*	*τ_2_ (ns)*	*<τ> (ns)*	*χ^2^*
***eGFP cytosol preparation***	0.179±0.005	1.55±0.02	0.821±0.005	2.73±0.02	2.60±0.04	1.024
***eGFP purified***	0.186±0.005	1.56±0.02	0.814±0.005	2.70±0.02	2.56±0.04	1.049

<τ> is the average lifetime calculated as described by equation (7). χ^2^ is the fit quality criterion calculated by the fitting software (see equation (6)). Errors are the 67% confidence intervals returned by the TRFA analysis software.

The results obtained for the purified eGFP and the cytosol preparation match within the confidence intervals and are in good agreement with previously reported values [40], [41], [42]. The excitation and emission spectra obtained from cytosol preparations of the two biosensors used in this work also match those from literature for CFP, Citrine and mTFP1 (data not shown) and, as discussed below, the fluorescence lifetime of mTFP that we obtained from a cytosol preparation is in good agreement with measurements by Visser et al. [29] on purified samples. We believe that these observations indicate that the cytosol preparation provides a reasonable analogue for measurements of purified fluorescent proteins. Our measurements of the fluorescence lifetime of ΔC11CFP are shorter than those reported in the literature for CFP, again see below, and we attribute this to the truncation of the 11 amino-acids from the protein.

### Fluorescence lifetime of the FRET donors: ΔC11CFP and mTFP

**Figure 2:**
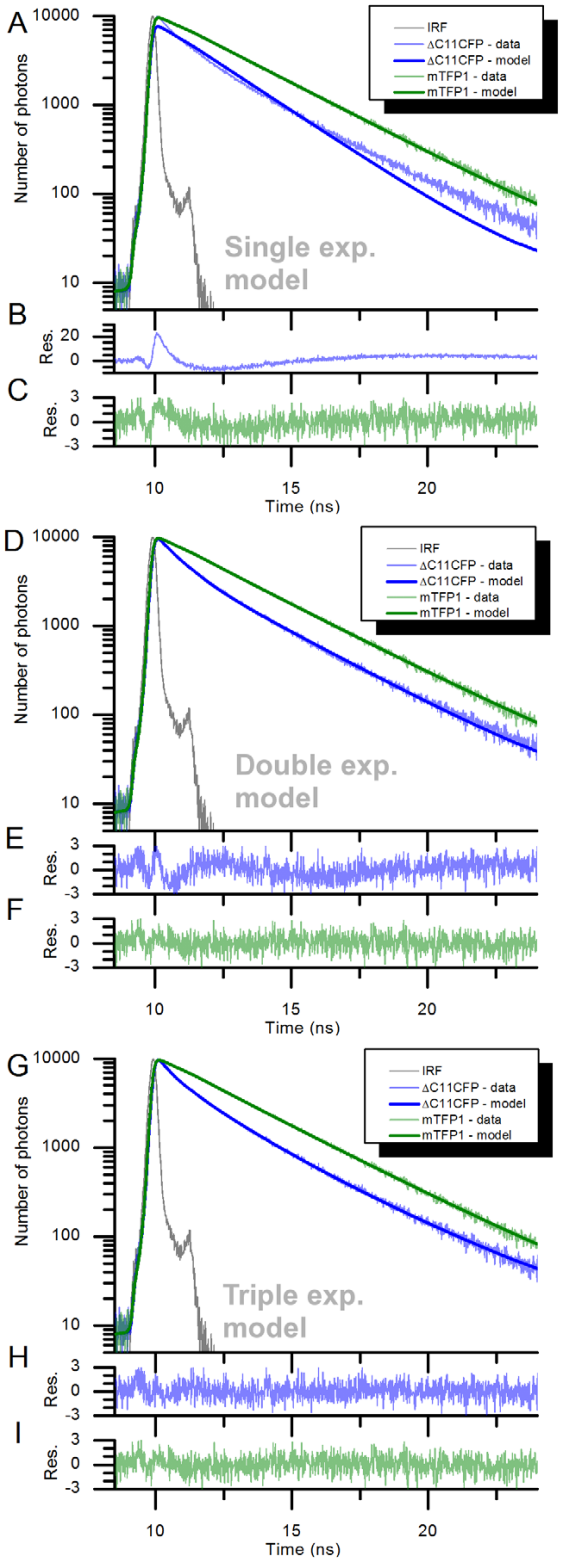
Fluorescence decays of ΔC11CFP and mTFP1 at 37°C and the fitted curve using a single exponential model (A), a double exponential model (D) and a triple exponential model (G). The respective residuals for ΔC11CFP, panels (B), (E) and (H), and mTFP, panels (C), (F) and (I), are shown underneath. IRF: Instrument Response Function. Res. : residuals.

The fluorescence lifetime of the donor fluorophores of TN-L15 and mTFP-TnC-Cit (respectively ΔC11CFP and mTFP1) were measured. **Figure 2** shows the fluorescence decays of ΔC11CFP and mTFP1 to which single, double and triple exponential decay models were fitted. ΔC11CFP was appropriately fitted (χ^2^ = 1.018) with a triple exponential model (as previously observed for ECFP [31]) although could be fitted with a reduced quality of fitting (χ^2^ = 1.374) with a double exponential model. The single exponential model does not describe well the decay of ΔC11CFP, as is highlighted by the poor fitting criterion (χ^2^ = 23.733) and the amplitude of the structure in the fitting residual (see panel B in **Figure 2**). For mTFP1 a double exponential leads to an excellent fit (χ^2^ = 1.006) but a reasonable fit can be obtained with a single exponential model (χ^2^ = 1.260). The attempt to fit a triple exponential model to the mTFP1 decay did not provide a further reduction in χ^2^ (χ^2^ = 1.052 for a triple exponential compared to χ^2^ = 1.006 for a double exponential).

The quality of the fit to the data can also be assessed by observing the residuals (**Figure 2** panels B, C, E, F, H and I). The residual curves obtained for ΔC11CFPusing the triple exponential model and mTFP1 double exponential model (panels H and F) are flat and do not exhibit any relevant features. This reveals that the fluorophores are best described by these models under the conditions used in this study.

However the simplified models tested (double exponential model for ΔC11CFP and single exponential model for mTFP1) lead to a reasonable fit (χ^2^<1.4) despite the presence of small features in the residuals (panels E and C) and a noticeable deviation of the fitted curve with respect to the data at late photon arrivals for the ΔC11CFP double exponential fit (panel D, between 20 ns and 24 ns). We note that the use of fluorophores with fewer decay components simplifies the analysis, particularly for FRET measurements, and has practical implications, for example with respect to lifetime changes caused by differential photobleaching of different components [43].

The parameters obtained from the three sets of fitting models are shown in Table 2. The results for mTFP1 are in reasonable agreement with earlier measurements from Visser et al. [29] who obtained *τ*_1_ = 0.91 ns, *p_1_* = 0.09 and *τ*_2_ = 2.9 ns, *p*_2_ = 0.91 carried out on purified proteins, or from Sun et al. [44], [45] who measured mTFP1 expressed in cells (2.73 ns and 2.68 ns). When fitting a double exponential model to mTFP1 the short lifetime component (1.3 ns) accounts only for 7% of the initial signal, which explains why the single exponential model leads to a reasonable fit.

**Table 2:** Fluorescence decay parameters obtained from the fitting of ΔC11CFP and mTFP1.

*FP*	*Model*	*p_1_*	*τ_1_ (ns)*	*p_2_*	*τ_2_ (ns)*	*p_3_*	*τ_3_ (ns)*	*<τ> (ns)*	*χ^2^*
***ΔC11CFP***	*Single*	*1*	*2.11±0.02*	*-*	*-*	*-*	*-*	*2.11*	*23.733*
	*Double*	*0.44±0.01*	*2.67±0.01*	*0.56*	*0.72±0.02*	*-*	*-*	*2.17*	*1.374*
	*Triple*	*0.27±0.01*	*3.01±0.05*	*0.34±0.01*	*1.59±0.05*	*0.40*	*0.50±0.04*	*2.19*	*1.018*
***mTFP***	*Single*	*1*	*2.75±0.01*	*-*	*-*	*-*	*-*	*2.75*	*1.260*
	*Double*	*0.93±0.01*	*2.81±0.01*	*0.07*	*1.3±0.3*	*-*	*-*	*2.76*	*1.006*
	*Triple*	*0.87*	*2.85±0.01*	*0.12±0.02*	*1.7±0.2*	*0.009±NA*	*2.5±1.9*	*2.76*	*1.052*

<τ> is the average lifetime calculated as described by equation (7). χ^2^ is the fit quality criterion calculated by the fitting software (see equation (6)). Errors are the 67% confidence intervals returned by the TRFA analysis software. FP: fluorescent protein.

The triple exponential fit of the ΔC11CFP decay shows that no decay component is predominant as each component (0.50 ns, 1.59 ns and 3.01 ns) has an approximately equal contribution. This is also observed in the double exponential fit where the two components (0.72 ns and 2.67 ns) approximately contribute the same amount to the decay.

We note that our measurement of the average fluorescence lifetime of ΔC11CFP (2.19 ns) is shorter than the published values, e.g. Geiger et al. 2.31 ns [31], Kim et al. 2.47 ns [46] and Borst et al. 2.71 ns [33]. These references show that there is significant variability in the fluorescence lifetime of CFP, which is presumably due to variations in the conditions of the measurement. However, given the magnitude and trend of the discrepancy between our data and the literature, we believe that at least part of this difference in measured lifetime is caused by the truncation of 11 amino-acids from the C-terminus of CFP.

Overall, these results highlight the complexity of the fluorescence decay of ΔC11CFP, which has at least two lifetime components of approximately equal contributions, and also demonstrate that mTFP1 can be reasonably well described by a single exponential model.

### Temperature dependence of mTFP1 and ΔC11CFP

It has been shown previously that the fluorescence lifetime of CFPs exhibits a significant dependence on temperature [25]. This property has the potential to affect the measurement of calcium concentration when using a sensor utilising CFP as a FRET donor.

**Figure 3:**
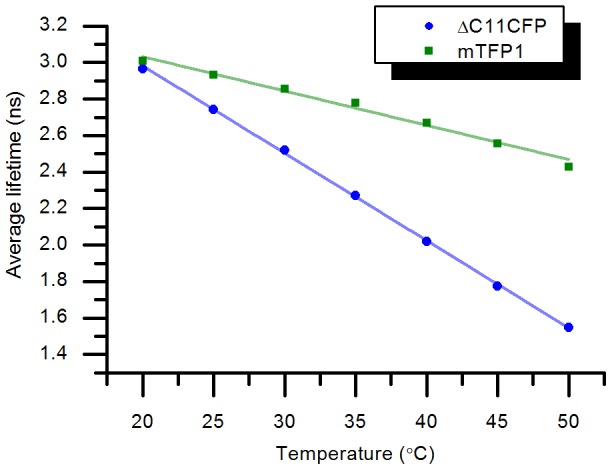
Temperature dependence of the average fluorescence lifetime of ΔC11CFP and mTFP1. A triple exponential model was used to describe ΔC11CFP fluorescence decays and a double exponential model for mTFP1. The average lifetimes were calculated using equation (7). Errors are the 67% confidence intervals returned by the TRFA analysis software. The straight lines represent the linear regression of the corresponding dataset with a slope of −0.048 ns/°C for ΔC11CFP and −0.019 ns/°C for mTFP1.

It is challenging to directly measure the effect of temperature on the calcium sensor fluorescence as the calcium buffers used to control the free calcium will also be affected by any changes in temperature, mostly through changes in dissociation constant of the calcium chelator (EGTA [35] is often used), and the calcium concentration would therefore not be controlled. However the direct effect of temperature on the donor fluorophore decay can be readily studied. We measured the fluorescence decays of ΔC11CFP and mTFP1 over the range 20°C to 50°C. A triple and a double exponential model were respectively used for ΔC11CFP and mTFP1, and the changes in average lifetime calculated using equation (7) are shown in **Figure 3**. We note that the acquisition times were adjusted so as to obtain 10,000 photons in the maximum of every fluorescence decay profile.

The average lifetime of ΔC11CFP follows an approximately linear relationship (R^2^ = 0.995) with temperature with a slope of −0.048 ns/°C, which is in good agreement with previous measurements by Villoing et al. [25]. Similarly the mTFP1 average lifetime is approximately linear with temperature with a slope of −0.019 ns/°C over the same range (R^2^ = 0.985). Therefore, the sensitivity of the average fluorescence lifetime with temperature is 2.5-fold lower for mTFP1 than for ΔC11CFP, which could improve the robustness of lifetime-based readouts of FRET biosensors using mTFP1 as a donor.

**Figure 4:**
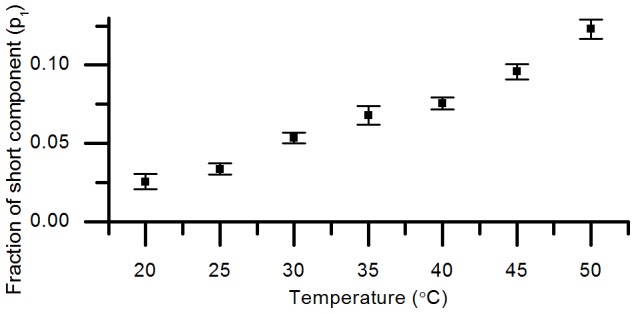
Fraction of short component (*p_1_*) versus temperature for mTFP1 when fitting a double exponential model. Errors are the 67% confidence intervals returned by the TRFA analysis software.

It is also useful to determine the extent to which mTFP1 can be reasonably described by a single exponential decay model as the temperature is varied. Figure 4 shows the initial fraction of the short decay component as a function of temperature when fitting a double exponential model to the mTFP1 temperature dependence dataset. The initial contribution of the short lifetime component (around 1 ns) is less than 2.6% at 20°C and increases with temperature but only exceeds 10% above 45°C. Based on the lifetime components given in Table 2, a 10% initial contribution in the short component represents only 5% of the total number of photons in the fluorescence decay (calculated from equation (8)) and at 37°C the short lifetime component contributes less than ≈3% of the emitted photons. Thus mTFP1 is well approximated to a single exponential decay model at 37°C and below. Changes with temperature in the relative contributions and lifetimes of ΔC11CFP and mTFP1 are described in the Figures S3 and S4.

### Fluorescence lifetime versus emission wavelength of mTFP1 and ΔC11CFP

**Figure 5:**
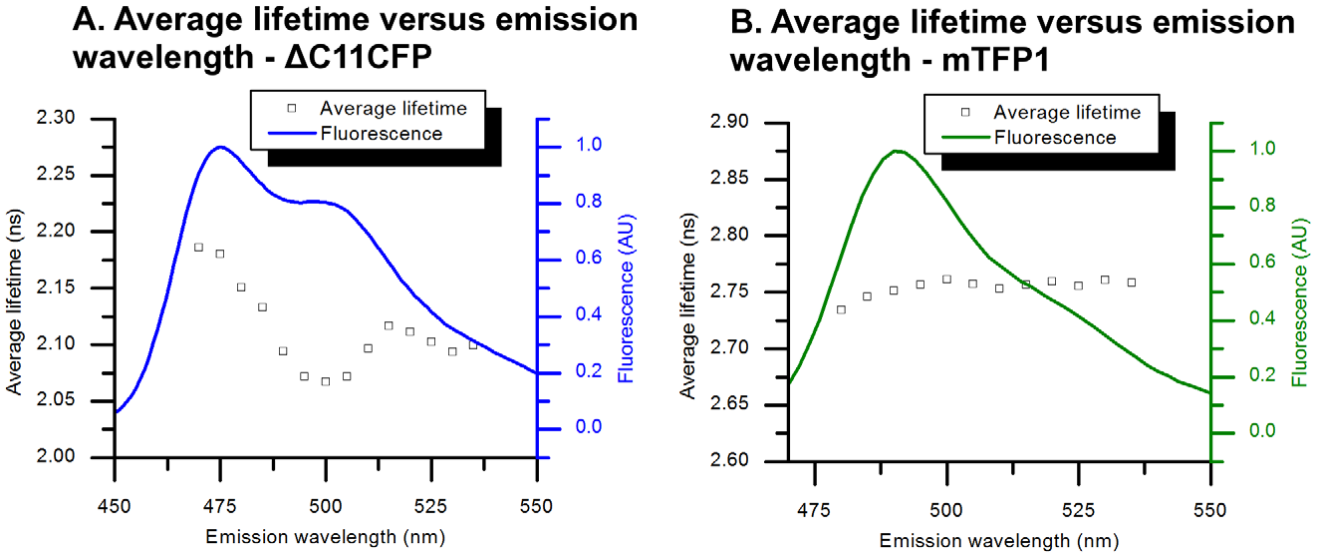
Emission wavelength and average fluorescence lifetime for (A) ΔC11CFP and (B) mTFP1. A triple exponential model was used to describe the ΔC11CFP fluorescence decays and a double exponential model for mTFP1. The average lifetimes were calculated using equation (7). The variation of all decay parameters with temperature are shown in figure S5 and S6.

The fluorescence lifetime of cyan variants of GFP has been shown previously to vary significantly with emission wavelength [25]. This means that changes in the choice of emission wavelength can affect the measured lifetime and complicate calculations of expected FRET efficiency. To our knowledge, the dependence of mTFP1 lifetime with emission wavelength has not been reported. Therefore, in order to perform a fair comparison, the dependence of both ΔC11CFP and mTFP1 fluorescence decays with the emission wavelength was investigated. A triple and a double exponential model were used for ΔC11CFP and mTFP1 respectively. **Figure 5** shows the changes in average fluorescence lifetimes (using equation (7)) of ΔC11CFP and mTFP1 with respect to their emission spectrum.

The average fluorescence lifetime of ΔC11CFP varies significantly across its emission spectrum with a maximum difference of 120 ps between 470 nm (2.19 ns) and 500 nm (2.07 ns). This significant change in average lifetime is a result of changes in fractional contributions of the two decay components as well as changes in the lifetimes of the two components, as shown in Figure S5 and S6 and previously reported by Villoing et al. [25].

The average fluorescence lifetime of mTFP1 across its emission spectrum is much more consistent than ΔC11CFP, with maximum difference of 27 ps between 480 nm (2.733 ns) and 500 nm (2.761 ns). This confers improved reliability and comparability between experiments using different emission channels and therefore between different instruments. This is important for translation of lifetime readouts, e.g. from solution phase-based experiments to fluorescence lifetime imaging (FLIM) microscopy where the instrumentation may have a much broader emission filter.

### Calcium titration analysed by global analysis of fluorescence lifetime data

In this set of experiments the donor fluorescence decay from TN-L15 and mTFP-TnC-Cit was measured at 12 different free calcium concentrations spanning from negligible calcium (≈0 µM) up to 40 µM. In the data analysis, two states of the biosensor were considered: one where it is in an “open” conformation (with, potentially, a low residual level of resonant energy transfer), expected to be predominant at low calcium levels and a second, in a closed conformation (where the FRET efficiency is higher). For both conformations, the population of donor fluorophores is assumed to have the same number of lifetime components (sub-populations) as the free donor fluorophore. Each dataset was fitted using global analysis, thereby allowing the determination of the lifetime components corresponding to each sub-population and their relative contributions at different calcium concentrations.

**Figure 6:**
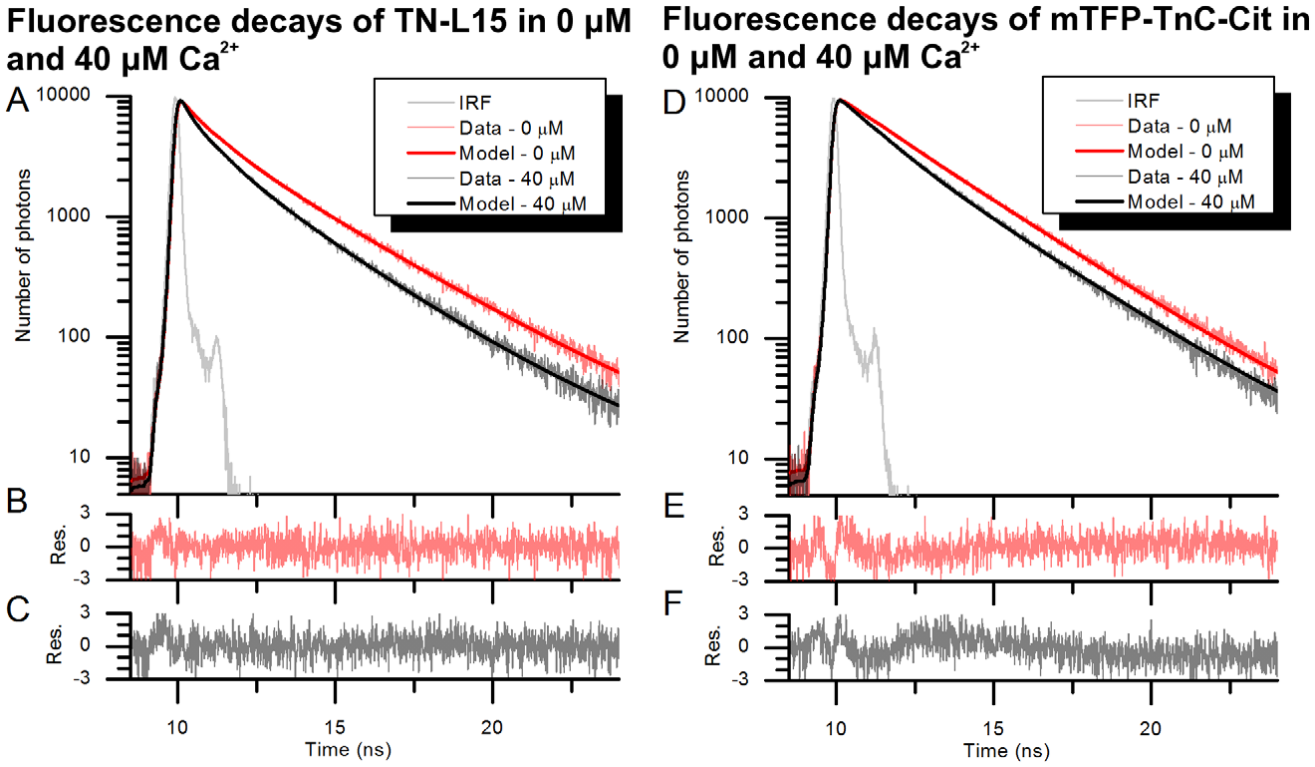
Fluorescence decays of TN-L15. **(A) and mTFP-TnC-Cit (D) in 0 µM and 40 µM of free calcium.** The resulting fit quality criteria (χ^2^) from the global fits are 1.128 and 1.316 for the TN-L15 and mTFP-TnC-Cit datasets respectively. (B) and (C) show the corresponding fit residuals at 0 µM and 40 µM for TN-L15. (E) and (F) show the fit residuals for mTFP-TnC-Cit at 0 µM and 40 µM. Res. : residuals.

Based on the results presented in **Table 2**, the free TN-L15 donor (i.e. ΔC11CFP) can be reasonably represented by a minimum of two decay components and so we choose to apply global analysis of TN-L15 based on a four-component model (i.e. two “high FRET” and two “low FRET” sub-populations). We note that global fitting of the TN-L15 titration dataset with a two-component model leads to an unacceptable fit with a χ^2^ = 1.773 (data not shown) and observe that a four-component model has previously been applied to describe a FRET system between CFP and YFP [47]. Since the mTFP1 fluorescence decay profile approximates well to a monoexponential decay profile, we choose to apply global analysis of mTFP-TnC-Cit using a two-component model. Figure 6 shows the fluorescence decay profiles, fitted curves and residuals obtained from the global analysis for TN-L15 and mTFP-TnC-Cit. For clarity, only the decay profiles corresponding to 0 µM and 40 µM are shown. The decay profiles displayed in Figure 6 panel A and D show the shortening in donor lifetime as the calcium level increases, as is expected due to FRET. The lifetime parameters obtained from the global analysis are shown in **Table 3**.

**Table 3:** Fluorescence lifetime results obtained from the global decay analysis of TN-L15 and mTFP-TnC-Cit.

Sensor	*Analysis*	*τ_1_ (ns)*	*τ_2_ (ns)*	*τ_3_ (ns)*	*τ_4_ (ns)*	*χ^2^*
***TN-L15***	4-components	*0.24±0.008*	*1.93±0.009*	*0.77±0.003*	*3.13±0.01*	1.128
		*Short – ‘closed’*	*Long – ‘closed’*	*Short – ‘open’*	*Long – ‘open’*	*-*
***TN-L15***	4-components+constant ratios ***r = 1.175*** **±0.007**	0.241±0.009	1.250±0.006	0.753±0.004	2.86±0.02	1.253
		*Short – ‘closed’*	*Long – ‘closed’*	*Short – ‘open’*	*Long – ‘open’*	*-*
***mTFP-TnC-Cit***	2-components	*2.601±0.002*	*1.114±0.006*	-	-	1.316
		*‘open’*	*‘closed’*	-	-	-

Each lifetime component is attributed to a conformation (low and high FRET, respectively equivalent to the open and closed conformation). For TN-L15 two different components are present in each population because a four-component model was used. Errors are the 67% confidence intervals returned by the TRFA analysis software.

**Figure 7:**
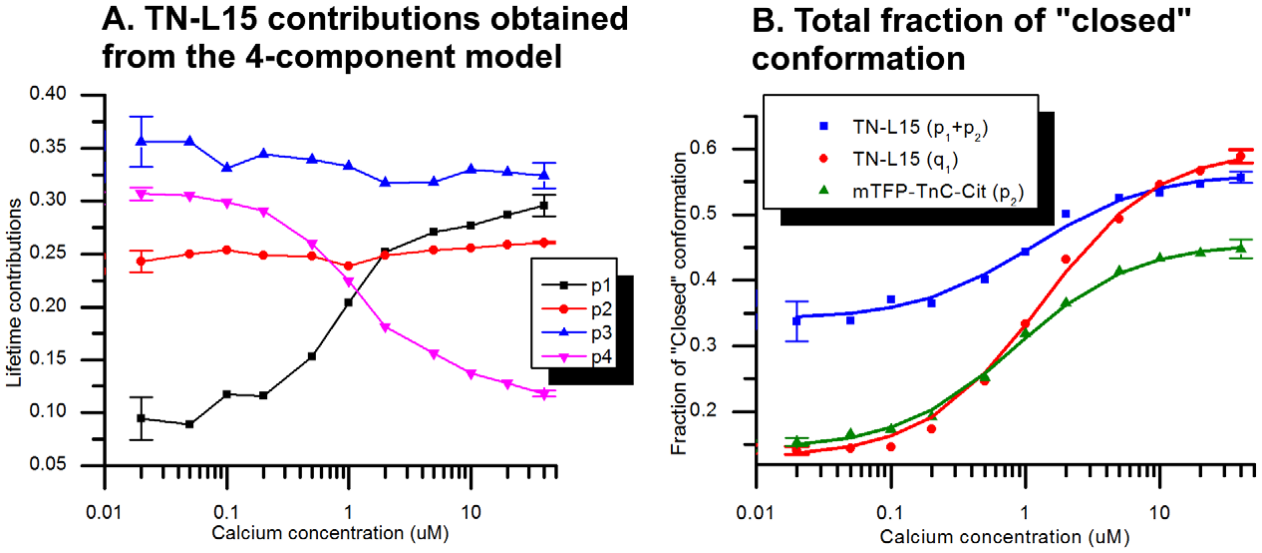
Fluorescence decay component contributions obtained from global analysis of the calcium titration data. (A) TN-L15 contributions from the 4-components model without constraints. (B) Total fraction of molecules in the closed conformation for TN-L15 (*p_2_+p_4_*) (4-components model), TN-L15 (*q_1_*) (4-component model with ratio constraint) and mTFP-TnC-Cit (*p_2_*) (2-component model), all fitted to equation (13) (solid lines). Error bars on the low and high calcium data points were calculated from three replicate decay acquisitions.

**Figure 7**A shows the contributions of the four lifetime components of TN-L15 as a function of calcium concentration. In this model, no assumptions about the relationships of contributions (*p_1_, p_2_, p_3_ and p_4_*) were made. The contributions *p_3_* and *p_4_* decrease with increasing calcium concentration, suggesting that they are associated with the “open” conformation that is assumed to be predominantly present at low calcium concentrations. Similarly the contributions *p_1_* and *p_2_* increase with increasing calcium concentration and we associate these with the “closed” conformation of the biosensor. The lifetimes *τ_3_* (0.77 ns) and *τ_4_* (3.13 ns) are then associated with the “open” conformation. We note however that both of these components are longer than those obtained from the double exponential fit of ΔC11CFP (0.72 ns and 2.67 ns, see **Table 2**). A possible explanation is that the fusion of CFP to the troponin C fragment affects the ΔC11CFP fluorescence lifetime. Unfortunately this means that it is not possible to determine the FRET efficiency of the TN-L15 biosensor in the two conformations since we cannot make a reasonable assumption about the values of the “non-FRETing” donor (or “donor only”) lifetimes.

The two component global fit of the mTFP-TnC-Cit data yielded lifetimes of *τ_1_* = 2.60 ns and *τ_2_* = 1.11 ns, which we attribute to the donor lifetimes of the “open” and “closed” biosensor conformations respectively. This is consistent with the changes observed in their relative contributions, *p_1_* and *p_2_* with increasing calcium concentration shown in Figure 7B. The donor lifetime of the open conformation is shorter than that obtained from free mTFP1 (2.75 ns, see Table 2), which can be reasonably attributed to a basal level of FRET under the assumption that the mTFP1 fluorescence decay profile is not affected by fusion to the Troponin C fragment, thereby allowing the use of equation (3). This yields FRET efficiencies of 5.3% (±0.1%) for the open conformation and 59.5% (±0.2%) for the closed conformation.

In order to relate the results from the global analysis of lifetime data to theory, we fitted the fraction of closed biosensor molecules to the titration model given in equation (13). The fraction of “closed” molecules is simply given by *p_2_* in the case of mTFP-TnC-Cit, whereas, for TN-L15, we used the total fraction of closed molecules (*p_1_+p_2_*). The titration model is defined by three parameters, i.e. *p_min_*, *p_max_* and *k_D_*, which were fitted to the data using a custom-built MATLAB® routine. The resulting curves are shown in Figure 7B and the fitted parameters are shown in Table 4. The dissociation constants, *k_D_*, obtained are in reasonable agreement with the reported value of 1.2 µM for TN-L15 [11]. Inspection of the fitted curves shown in Figure 7B indicates that the data from mTFP-TnC-Cit (green) provides a more robust fitting to the titration model than the four-component global fitting of the TN-L15 lifetime data (blue).

**Table 4:** Titration model parameters.

	*Analysis*	*k_D_ (µM)*	*p_min_*	*p_max_*	*Error @* [*Ca^2+^*]* = 1 µM*
***TN-L15***	4-components	1.12	0.34	0.56	0.4 *µM*
***TN-L15***	4-components+constant ratios ***r = 1.175*** **±0.007**	1.32	0.13	0.6	0.1 *µM*
***mTFP-TnC-Cit***	2-components	0.86	0.14	0.46	0.1 *µM*
***mTFP-TnC-Cit***	Phasor plot	1.04	0.20	0.44	0.1 *µM*

These parameters are obtained from fitting the theoretical titration model to the dataset. The error on the concentration measured at [*Ca^2+^*]* = 1 µM* is shown in the last column on the right.

We can use the fitted parameters from the titration data to estimate the accuracy to which calcium concentrations can be determined from fluorescence lifetime measurements of these two FRET biosensors. If we fix the titration parameters (*p_min_*, *p_max_* and *k_D_*) to the values obtained from the global analysis, we can calculate how the error on the measurement of the fraction of donor population undergoing FRET impacts the estimation of calcium concentration using equation (14). For these calculations we considered the inflexion point (here at [*Ca^2+^*]* = 1 µM*) where the sensitivity of the change in the fraction of the donor population undergoing FRET with calcium concentration is maximal and approximately linear. The error on the population is taken as the average of the experimental errors of the two end points (at 0 and 40 µM). The resulting uncertainty in calcium concentration is found to be 0.4 µM for the TN-L15 biosensor and 0.1 µM for the mTFP-TnC-Cit biosensor.

**Figure 8:**
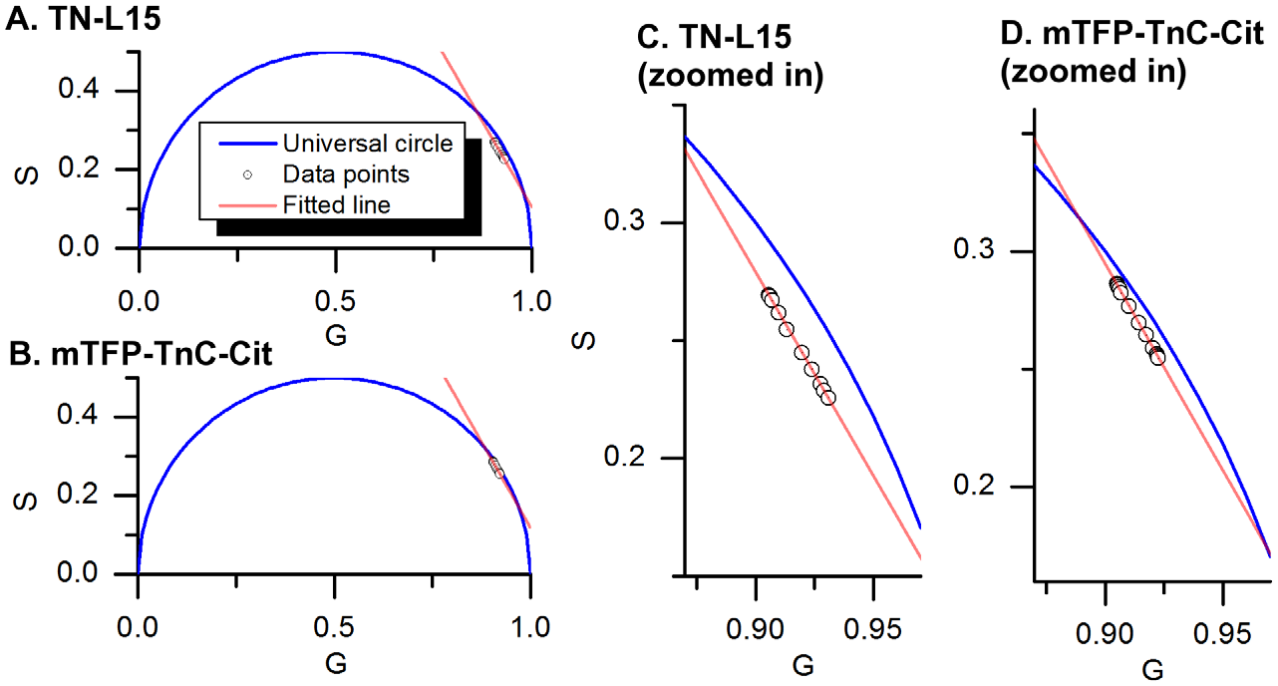
Phasor plots of the titration dataset obtained from TN-L15 (panels A and C) and mTFP-TnC-Cit (panels B and D). The panels C and D are close-ups of the region of the phasor plot containing the data-points. The green circle represents the Rhodamine 6G dataset and lies on the universal circle with a lifetime of 4.01 ns. Each black circle represents a fluorescence decay measured at a particular calcium level. The red line shows the linear fit across all data-points and crossing the universal circle (red circles).

From **Figure 7**A we note, however, that the contributions from the two donor sub-populations (long and short lifetime component) within the same conformation (i.e. p_2_ and p_1_ in the “closed” conformation and p_4_ and p_3_ in the “open” conformation) do not vary in proportion across the titration, as could be expected since the two CFP components is a result of a slow equilibrium between two states of the chromophore [48]. To investigate this further we applied the phasor analysis (polar plot) [36] to the titration data sets, as discussed in the materials and methods section. For this the titration dataset is represented by a set of points on the phasor plot as shown in **Figure 8** (black circles). It is immediately clear that these points map to a straight line (red line, **Figure 8**) as is expected for a mixture of “low FRET” and “high FRET” populations of donor fluorophore.

**Figure 9:**
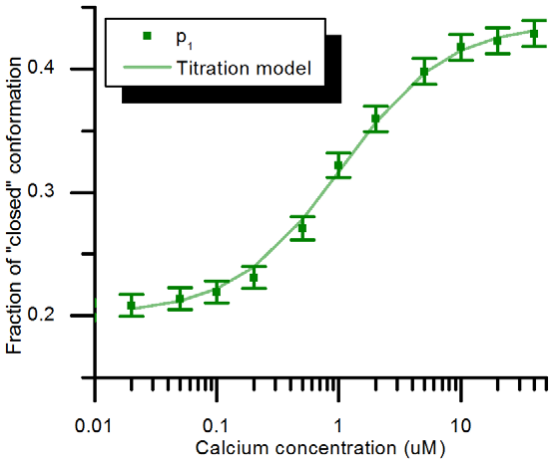
Fraction of closed conformation obtained from the phasor plot analysis of mTFP-TnC-Cit. The lifetimes calculated from the phasor plot are 2.80 ns for the open conformation and 1.43 ns for the closed conformation. The error bars were obtained from experimental repeats.

For mTFP-TnC-Cit, the near mono-exponential character of mTFP1 suggests that the phasor plot could be interpreted as a chord between two phasor points on the universal circle that correspond to the two pure components, i.e. (“open” and “closed” conformations). It is therefore possible to obtain the lifetimes of both conformations (intersects between the line and the universal circle) and the fraction of each conformation at each data-point. The lifetimes obtained from this method are 1.43 ns±0.02 ns and 2.81 ns±0.01 ns and the fractions of “closed” conformation calculated at each point of the titration are shown in **Figure 9**. Fitting the titration model to this dataset leads to a dissociation constant of 1.04 µM, *p_min_ = 0.20* and *p_max_ = 0.44*, which are in reasonable agreement with those obtained from the global fitting (**Table 4**). The lifetime values obtained with this method are both longer than those that were obtained by global analysis fitting (2.601 ns±0.006 ns and 1.114 ns±0.006 ns). This discrepancy may result from the assumptions of our fitting model, (e.g. the monoexponential decay profile of mTFP).

For TN-L15, because of the multi-exponential decay profiles of CFP, it is not possible to identify the positions of the pure “open” and “closed” conformations of the TN-L15 sensor from the phasor plot analysis. In fact these could lie anywhere between the extreme points of the titration data along the fitted line and its intersection with the universal circle. Thus it is not possible to determine the fractions of each conformation for the TN-L15 biosensor, without resorting to the use of phasor plots calculated at higher harmonics.

However, the observation that the phasor points lie on a straight line indicates that the “low FRET” and “high FRET” populations have a constant phasors (a constant fluorescence decay signature) and therefore the ratio p_2_/p_1_ and p_4_/p_3_ remain fixed, under our assumption that their respective lifetimes are invariant. We can then utilize this information to modify the four-component model used for TN-L15. Accordingly, we fitted the TN-L15 titration data to a constrained 4-component model, described by equation (9), in which the two ratios (long to short lifetime contribution in both the “open” and “closed” conformation) are linked and globally fitted, resulting into a single ratio parameter (*r_21_ = r_43_ = r*) for the whole titration dataset. The quality of fit (χ^2^ = 1.253) is similar to that obtained with the four-component model without constraints (χ^2^ = 1.128), suggesting that the dataset is well described by this constrained 4-component model. The lifetime components obtained from this constrained global fitting are shown in **Table 3**, and the variation of the total contribution of the closed conformations (*q_1_* as described by equation (10)) with calcium concentration are plotted in **Figure 7**B. The “low FRET” and “high FRET” populations are described respectively by 2.86 ns±0.02 ns/0.753 ns±0.004 ns (54%/46%) and 1.250 ns±0.006 ns/0.241 ns±0.009 ns (54%/46%).

**Table 4** presents the results obtained from fitting the titration model to the total fraction of “closed” conformations (*q_1_*). The dissociation constant (1.32 µM) is in good agreement with that previously published [11]. We note that this constrained ratio model results in a larger dynamic range of the TN-L15 sensor with the fraction of “closed” conformation now changing from 13% at low calcium to 60% at high calcium.

### Calcium titration analysed with respect to single-exponential model donor lifetime

**Figure 10:**
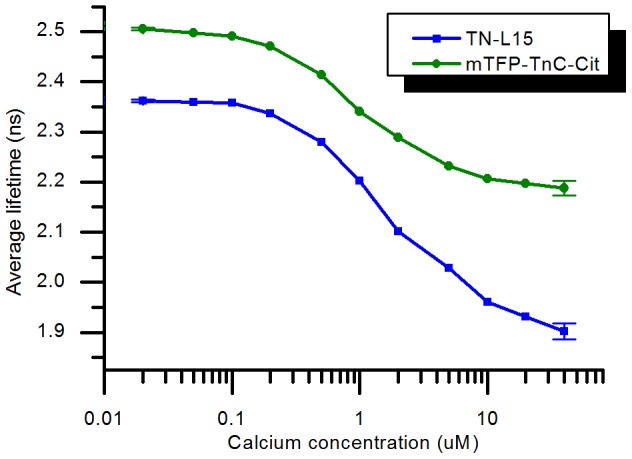
TN-L15 and mTFP-TnC-Cit fluorescence lifetimes obtained from a single exponential fit. The fitting criteria are χ^2^ = 22.867 for TN-L15 and χ^2^ = 4.569 for mTFP-TnC-Cit. The error bars show the standard deviation calculated from three replicate decay acquisitions.

The titration data can also be visualized by plotting the changes in donor fluorescence lifetime (obtained by fitting a single exponential decay model to the data) with increasing calcium concentration, as shown in **Figure 10**. This is common practice for FLIM experiments where insufficient photons are detected to accurately fit a multi-exponential decay model on a pixel by pixel basis and global fitting software is not available. For the data in **Figure 10** the total number of photons acquired for each fluorescence decay profile measurement was approximately constant as the acquisition for each was stopped when the number of photons in the peak of the decay reached 10,000 and CFP and mTFP1 have similar fluorescence lifetimes. The lifetime of CFP is seen to vary from 2.36 ns for negligible calcium to 1.90 ns at 40 µM of calcium, exhibiting a total change in lifetime of 460 ps, while for mTFP1 the lifetime changes from 2.51 ns to 2.18 ns, i.e. a 330 ps lifetime change. The graphs in **Figure 10** can thus be used to build a calibration curve and permit measurements of calcium concentration using average fluorescence lifetimes obtained by fitting time-resolved data to a single exponential decay model.

It is also possible to estimate the error on the calcium concentration determination from the single exponential fit around the inflexion point (at 1 µM calcium). For this, a three-point slope estimation was used to calculate the local slope and the average error from the end points (0 and 40 µM calcium) was used to estimate the error on the lifetime at 1 µM. The calculation leads to similar errors for both TN-L15 and mTFP-TnC-Cit of 0.1 µM under these measurement conditions.

### Live-cell FLIM experiment of TN-L15 and mTFP-TnC-Cit

**Figure 11:**
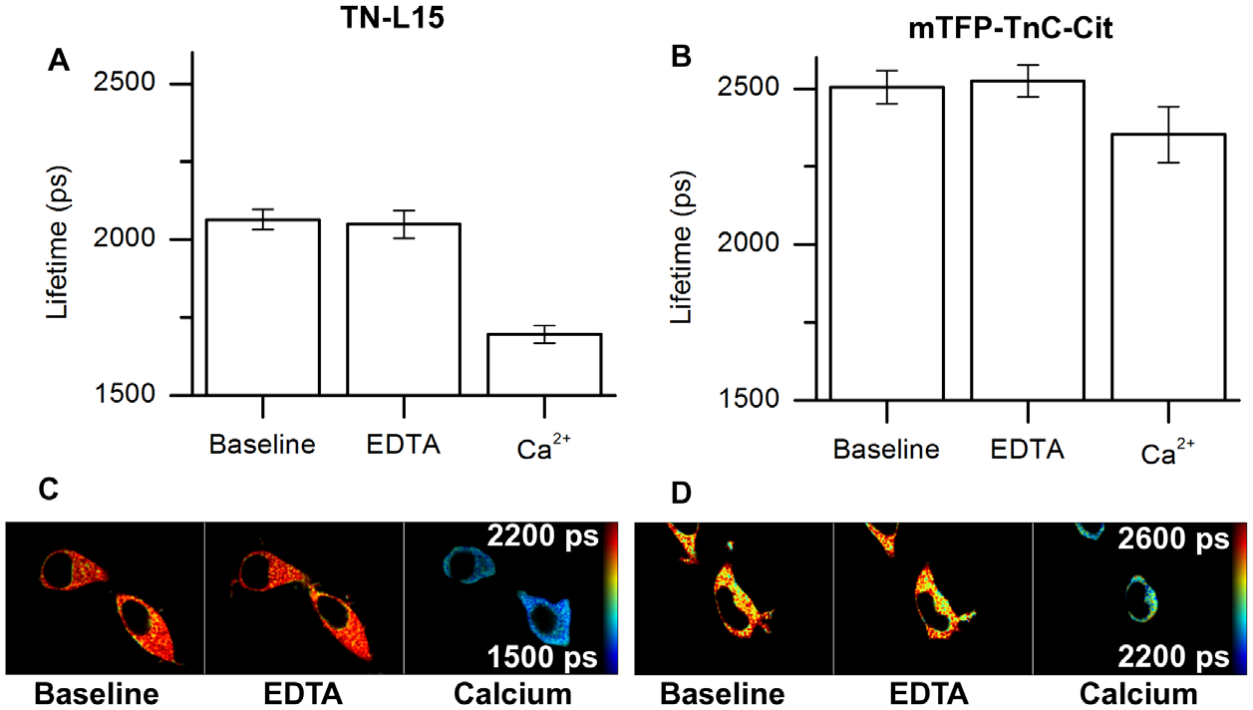
Fluorescence lifetime imaging of HEK293T cells transiently transfected with either TN-L15 or mTFP-TnC-Cit. (A) Fluorescence lifetime (obtained from single exponential model fitting) of TN-L15 before EDTA exposure (baseline: 2060 ps±30 ps), after exposure to 10 mM EDTA/5 µM ionomycin (EDTA: 2050 ps±40 ps) and after calcium stimulation (Ca^2+^: 1700 ps±30 ps) (N = 4). (B) Fluorescence lifetime of mTFP-TnC-Cit before EDTA exposure (baseline: 2510 ps±50 ps), after exposure to 10 mM EDTA/5 µM ionomycin (EDTA: 2520 ps±50 ps) and after calcium stimulation (Ca^2+^: 2350 ps±90 ps) (N = 4). (C) FLIM images of TN-L15. (D) FLIM images of mTFP-TnC-Cit.

Live-cell FLIM experiments were carried out in HEK293T cells transiently transfected with either TN-L15 or mTFP-TnC-Cit. The results are shown in **Figure 11**. In cells transfected with TN-L15 the fluorescence lifetime of CFP was 2060 ps±30 ps and remained approximately constant (2050 ps±40 ps) after EDTA/ionomycin exposure but decreased to 1700 ps±30 ps after calcium stimulation (**Figure 11**, panel A and C). The same experimental protocol with cells expressing mTFP-TnC-Cit showed that the fluorescence lifetime of mTFP1 did not change significantly between the baseline condition (2510 ps±50 ps) and the EDTA/ionomycin exposure (2530 ps±50 ps) and decreased to 2350 ps±90 ps after calcium stimulation (**Figure 11**, panel B and D). With both calcium sensors, since the lifetimes are similar between the baseline in calcium-free medium and the EDTA/ionomycin exposure we conclude that there was no calcium release from the endoplasmic reticulum stores upon EDTA exposure.

The calcium stimulation thus caused the lifetime of both donor fluorophores to decrease, as expected when FRET occurs. However, the fluorescence lifetimes measured from TN-L15 in live-cells are inconsistent with those measured in solution phase (2360 ps at low calcium to 1900 ps at high calcium, see **Figure 10**). This is may be due to the sensitivity of CFP lifetime to its environment and measurement conditions (i.e. cytosol preparation compared to cytosol in a live cell, and to different emission wavelength and broader spectral band-pass detection in the FLIM microscope). For the TN-L15 sensor, it is therefore challenging to establish absolute measurements of calcium concentration using the calibration parameters obtained from the solution phase analysis. The fluorescence lifetime of mTFP-TnC-Cit measured in live-cells under low calcium conditions (2510 ps) is in agreement with the measurements from the cytosol preparation (2511 ps). The fluorescence lifetime (2350 ps) of cells at high calcium concentration is higher than that measured under high calcium concentrations in the cytosol preparation (2188 ps) and further work is required to explain this difference.

## Discussion

We have shown that both calcium biosensors achieve a similar accuracy in determination of calcium concentration when the fluorescence decay profile is analysed with a single exponential decay model. This approach, presented in Figure 10, is representative of the data analysis procedure usually used for imaging (FLIM), where the number of photons available is normally insufficient to fit a more complex decay model. Here, the slightly higher precision in lifetime determination obtained for the mTFP-TnC-Cit sensor is counter-balanced by the larger change in fluorescence lifetime of the TN-L15 sensor. For this approach to be used to determine calcium concentrations, e.g. FLIM measurements in cells, it is necessary to first establish a calibration curve such as shown in Figure 10, which may not always be convenient or possible.

It would be useful to establish the extent to which the fusion of donor fluorophores to the troponin C fragment affects their fluorescence decay profiles. It seems likely that this is a particular issue for CFP - as evidenced by the longer lifetime seen for the open construct compared to CFP alone. The sensitivity of the fluorescence lifetime of ECFP to the fusion to a Troponin C-like protein was previously observed [31], where the fusion protein TN-XXL ΔcpCit (the TN-XXL protein from which the acceptor fluorophore was truncated) exhibits a fluorescence lifetime of about 100 ps longer than that of ECFP. In principle, this could be investigated for our TN-L15 biosensor through the development of further control constructs, e.g. removing the acceptor fluorophore from the biosensor, incorporating a non-fluorescent acceptor fluorophore such as Amber fluorescent protein [49], [50], or introducing an “Amber-like” mutation preventing the formation of the fluorophore [31].

The dependence of ΔC11CFP fluorescence lifetime with temperature (−0.048 ns/°C) is 2.5-fold that of mTFP1 (−0.019 ns/°C) making lifetime measurements of mTFP1 less subject to temperature artefacts. Also mTFP1 fluorescence lifetime does not vary significantly with emission wavelength, unlike ΔC11CFP that exhibits a lifetime shift of 120 ps over its emission spectrum. This could be important for FLIM microscopy and other applications where relatively broad emission filters are employed. And, as demonstrated by Padilla-Parra et al. [51], the fluorescence lifetime of mTFP1 is much less sensitive to photobleaching than CFP, which can undergo photoconversion [43]. Therefore, the reduced sensitivity of mTFP1 to its environment makes it a more robust sensor for quantitative fluorescence lifetime measurements.

In general, when considering quantitative fluorescence readouts based on the analysis of fluorescence decay profiles, e.g. FRET assays of interacting population fractions or time-resolved anisotropy measurements, a more complex fitting model can be used to directly provide quantitative data, although it is necessary to assume an appropriate fitting model *a priori*. In the case of CFP-based donors, this fitting entails the determination of at least four lifetime components (two “high FRET” and “low FRET” components) whereas the use of mTFP1 permits a simpler analysis requiring only two decay components. In FLIM experiments it is usually not practical to fit even a two exponential decay on a pixel-wise basis and so the lifetimes of the fluorescence decay components must be either determined in advance and fixed or determined globally during the image analysis. In either case, mTFP1 is advantageous as it requires fewer lifetime components to be determined.

For multi-exponential decay analysis of the TN-L15 and mTFP-TnC-Cit biosensors, the knowledge of *p*_min_, *p*_max_ and *k_D_*, is also required for measurements of absolute calcium concentration, here being obtained through high precision solution phase measurements. For FLIM experiments mapping calcium concentration, global fitting of the donor fluorescence decay profiles could directly indicate variations in calcium concentration and, if absolute calcium concentrations are required, these may be estimated from globally fitted lifetime data using the *p*_min_, *p*_max_ and *k_D_* values reported here.

For the unconstrained four-component global fit to the TN-L15 data (**Figure 7**A), the relative contributions of the four components do not vary proportionately as expected, i.e. the ratio of the pre-exponential factors attributed to the open conformation (*p*_4_/*p*_3_) (and that attributed to the closed conformation *p_2_/p_1_*) is not constant with varying calcium concentration. This could be an artefact resulting from the difficulty in accurately determining the contributions of the four decay components and/or the fact that CFP exhibits a fluorescence decay profile that is more complex than the double exponential model assumed for the sensor. The constrained global fitting model used to analyse TN-L15 data set inherently addresses this issue by globally fitting the ratios of the two components (long to short lifetime component). The ratio is then constant across the titration. This model is supported by the results obtained from the phasor analysis, in which the data points lie on a straight line, for both TN-L15 and mTFP-TnC-Cit, suggesting that the open and closed conformations have different but fixed decay signatures.

Under conditions of negligible calcium when the biosensors are expected to be in the open conformation, the fraction attributed to the closed conformation is still 13% for the TN-L15 sensor and 15% for mTFP-TnC-Cit. This could be explained by issues during protein maturation causing some biosensor molecules to be permanently locked into a closed conformation or dimerization of the probe in the open conformation leading to inter-molecular FRET. Another explanation could be that the donor and acceptor present a distribution of donor-acceptor distances and/or relative dipole orientations and so the assumption of a unique FRET efficiency for the open conformation that underlies our fitting model is incorrect. Similarly, we note that not all biosensors appear to be closed in the high calcium range (40 µM). This can be explained by the possible protonation of the Citrine at pH = 7.2 [31]. The work by Geiger et al. reported a 15% of protonated Citrine at pH = 7.2, 23°C. The higher temperature used in our experiments (37°C) may explain a higher fraction of protonated Citrine. However we cannot rule out the possibility of incomplete maturation of the acceptor fluorophore, the presence of misfolded non-functional molecules (caused by overexpression of the fluorescent protein) that are incapable of closing, as already discussed by Padilla-Parra et al. [51], or the impact of a distribution of donor-acceptor distances or fluorophore orientations when the biosensor is in the closed conformation.

From the imaging experiments we conclude that the difference between the lifetimes obtained for TN-L15 in solution phase and in live cells, which we attribute to the sensitivity of CFP lifetime to its environment and to measurement conditions, make the absolute calcium measurements challenging with TN-L15 in live cells. mTFP-TnC-Cit, however, presented more similar lifetimes in each environment and should be more useful for quantitative measurements.

In conclusion, a new calcium FRET biosensor, mTFP-TnC-Cit, was constructed by replacing the CFP fluorophore within TN-L15 with mTFP1. Since mTFP1 fluorescence decay can be well described by a single exponential decay profile, this FRET biosensor fits well to a two-component donor fluorescence decay model for which fitting and analysis are relatively straightforward. Furthermore, mTFP1 is also significantly less sensitive to changes in temperature and emission wavelength compared to CFP and we therefore suggest that it is more suitable for many FLIM experiments than CFP-based probes. We note, however, that many existing biosensors, such as the TN-L15 FRET biosensor, do incorporate CFP and we have shown that a constrained four-component model of CFP-based FRET can give a good description of the system, and can be supported by phasor analysis. We also conclude that the TN-L15 and mTFP-TnC-Cit can lead to a similar precision in calcium determination as long as long as an appropriate model is used. We also note that our titration experiment suggests that a similar precision in determination of calcium concentration can be achieved with both these FRET biosensors when fitting only a single exponential donor fluorescence decay model to the data.

## Supporting Information

Figure S1 **Sequences of the forward and reverse primers for the creation of mTFP-TnC-Cit from TN-L15 and pmTFP-N.**
(PDF)

Figure S2 **Sequence of forward and reverse primers used for the creation of ΔC11CFP.** The Ochre STOP codon is shown in red.
(PDF)

Figure S3 **Temperature dependence of ΔC11CFP decay components (left) and contributions (right).** All decays were fitted with a triple exponential model.
(TIF)

Figure S4 **Temperature dependence of mTFP1 decay components (left) and contributions (right).** All decays were fitted with a double exponential model.
(TIF)

Figure S5 **ΔC11CFP decay components (left) and their contributions (right) at different emission wavelength.** All decays were fitted with a triple exponential model.
(TIF)

Figure S6 **mTFP1 decay components (left) and their contributions (right) at different emission wavelength.** All decays were fitted with a double exponential model.
(TIF)

## References

[1] Clapham DE (1995) CALCIUM SIGNALING. Cell 80: 259–268. 7834745 10.1016/0092-8674(95)90408-5

[2] Berridge MJ, Lipp P, Bootman MD (2000) The versatility and universality of calcium signalling. Nature Reviews Molecular Cell Biology 1: 11–21. 11413485 10.1038/35036035

[3] Carafoli E (2004) Calcium-mediated cellular signals: a story of failures. Trends in Biochemical Sciences 29: 371–379. 15236745 10.1016/j.tibs.2004.05.006

[4] Mank M, Griesbeck O (2008) Genetically encoded calcium indicators. Chemical Reviews 108: 1550–1564. 18447377 10.1021/cr078213v

[5] Nakai J, Ohkura M, Imoto K (2001) A high signal-to-noise Ca2+ probe composed of a single green fluorescent protein. Nature Biotechnology 19: 137–141. 10.1038/84397 11175727

[6] Nagai T, Sawano A, Park ES, Miyawaki A (2001) Circularly permuted green fluorescent proteins engineered to sense Ca2+. Proceedings of the National Academy of Sciences of the United States of America 98: 3197–3202. 11248055 10.1073/pnas.051636098 PMC30630

[7] Zhao YX, Araki S, Jiahui WH, Teramoto T, Chang YF, et al (2011) An Expanded Palette of Genetically Encoded Ca2+ Indicators. Science 333: 1888–1891. 21903779 10.1126/science.1208592 PMC3560286

[8] Tang S, Wong HC, Wang ZM, Huang Y, Zou J, et al (2011) Design and application of a class of sensors to monitor Ca2+ dynamics in high Ca2+ concentration cellular compartments. Proceedings of the National Academy of Sciences of the United States of America 108: 16265–16270. 21914846 10.1073/pnas.1103015108 PMC3182728

[9] Miyawaki A, Llopis J, Heim R, McCaffery JM, Adams JA, et al (1997) Fluorescent indicators for Ca2+ based on green fluorescent proteins and calmodulin. Nature 388: 882–887. 9278050 10.1038/42264

[10] Garaschuk O, Griesbeck O, Konnerth A (2007) Troponin C-based biosensors: A new family of genetically encoded indicators for in vivo calcium imaging in the nervous system. Cell Calcium 42: 351–361. 17451806 10.1016/j.ceca.2007.02.011

[11] Heim N, Griesbeck O (2004) Genetically encoded indicators of cellular calcium dynamics based on troponin C and green fluorescent protein. J Biol Chem 279: 14280–14286. 14742421 10.1074/jbc.M312751200

[12] Mank M, Reiff DF, Heim N, Friedrich MW, Borst A, et al (2006) A FRET-based calcium biosensor with fast signal kinetics and high fluorescence change. Biophys J 90: 1790–1796. 16339891 10.1529/biophysj.105.073536 PMC1367327

[13] Griesbeck O, Baird GS, Campbell RE, Zacharias DA, Tsien RY (2001) Reducing the environmental sensitivity of yellow fluorescent protein - Mechanism and applications. Journal of Biological Chemistry 276: 29188–29194. 11387331 10.1074/jbc.M102815200

[14] Lakowicz JR (2006) Pinciples of Fluorescence Spectroscopy. NY: Springer.

[15] Ting AY, Kain KH, Klemke RL, Tsien RY (2001) Genetically encoded fluorescent reporters of protein tyrosine kinase activities in living cells. Proceedings of the National Academy of Sciences of the United States of America 98: 15003–15008. 11752449 10.1073/pnas.211564598 PMC64973

[16] Markova O, Mukhtarov M, Real E, Jacob Y, Bregestovski P (2008) Genetically encoded chloride indicator with improved sensitivity. Journal of Neuroscience Methods 170: 67–76. 18279971 10.1016/j.jneumeth.2007.12.016

[17] Becker W, Bergmann A, Hink MA, Konig K, Benndorf K, et al (2004) Fluorescence lifetime imaging by time-correlated single-photon counting. Microscopy Research and Technique 63: 58–66. 14677134 10.1002/jemt.10421

[18] Wouters FS, Bastiaens PIH (1999) Fluorescence lifetime imaging of receptor tyrosine kinase activity in cells. Current Biology 9: 1127–1130. 10531012 10.1016/s0960-9822(99)80484-9

[19] Tramier M, Gautier I, Piolot T, Ravalet S, Kemnitz K, et al (2002) Picosecond-hetero-FRET microscopy to probe protein-protein interactions in live cells. Biophysical Journal 83: 3570–3577. 12496124 10.1016/S0006-3495(02)75357-5 PMC1302432

[20] Berney C, Danuser G (2003) FRET or no FRET: A quantitative comparison. Biophysical Journal 84: 3992–4010. 12770904 10.1016/S0006-3495(03)75126-1 PMC1302980

[21] Suhling K, French PMW, Phillips D (2005) Time-resolved fluorescence microscopy. Photochemical & Photobiological Sciences 4: 13–22. 15616687 10.1039/b412924p

[22] Heim R, Tsien RY (1996) Engineering green fluorescent protein for improved brightness, longer wavelengths and fluorescence resonance energy transfer. Current Biology 6: 178–182. 8673464 10.1016/s0960-9822(02)00450-5

[23] Rizzo MA, Springer GH, Granada B, Piston DW (2004) An improved cyan fluorescent protein variant useful for FRET. Nature Biotechnology 22: 445–449. 10.1038/nbt945 14990965

[24] Bourett TM, Sweigard JA, Czymmek KJ, Carroll A, Howard RJ (2002) Reef coral fluorescent proteins for visualizing fungal pathogens. Fungal Genetics and Biology 37: 211–220. 12431456 10.1016/s1087-1845(02)00524-8

[25] Villoing A, Ridhoir M, Cinquin B, Erard M, Alvarez L, et al (2008) Complex Fluorescence of the Cyan Fluorescent Protein: Comparisons with the H148D Variant and Consequences for Quantitative Cell Imaging. Biochemistry 47: 12483–12492. 18975974 10.1021/bi801400d

[26] Yasuda R, Harvey CD, Zhong HN, Sobczyk A, van Aelst L, et al (2006) Supersensitive Ras activation in dendrites and spines revealed by two-photon fluorescence lifetime imaging. Nature Neuroscience 9: 283–291. 16429133 10.1038/nn1635

[27] Goedhart J, van Weeren L, Hink MA, Vischer NOE, Jalink K, et al (2010) Bright cyan fluorescent protein variants identified by fluorescence lifetime screening. Nature Methods 7: 137–U174. 20081836 10.1038/nmeth.1415

[28] Ai HW, Henderson JN, Remington SJ, Campbell RE (2006) Directed evolution of a monomeric, bright and photostable version of Clavularia cyan fluorescent protein: structural characterization and applications in fluorescence imaging. Biochemical Journal 400: 531–540. 16859491 10.1042/BJ20060874 PMC1698604

[29] Visser A, Laptenok SP, Visser NV, van Hoek A, Birch DJS, et al (2010) Time-resolved FRET fluorescence spectroscopy of visible fluorescent protein pairs. European Biophysics Journal with Biophysics Letters 39: 241–253. 19693494 10.1007/s00249-009-0528-8

[30] Day RN, Booker CF, Periasamy A (2008) Characterization of an improved donor fluorescent protein for Forster resonance energy transfer microscopy. Journal of Biomedical Optics 13. 10.1117/1.2939094 PMC2483694 18601527

[31] Geiger A, Russo L, Gensch T, Thestrup T, Becker S, et al (2012) Correlating Calcium Binding, Forster Resonance Energy Transfer, and Conformational Change in the Biosensor TN-XXL. Biophysical Journal 102: 2401–2410. 22677394 10.1016/j.bpj.2012.03.065 PMC3353025

[32] McGinty J, Stuckey DW, Soloviev VY, Laine R, Wylezinska-Arridge M, et al (2011) In vivo fluorescence lifetime tomography of a FRET probe expressed in mouse. Biomedical optics express 2: 1907–1917. 21750768 10.1364/BOE.2.001907 PMC3130577

[33] Borst JW, Laptenok SP, Westphal AH, Kuhnemuth R, Hornen H, et al (2008) Structural changes of yellow Cameleon domains observed by quantitative FRET analysis and polarized fluorescence correlation spectroscopy. Biophys J 95: 5399–5411. 18790855 10.1529/biophysj.107.114587 PMC2586569

[34] Manning HB, Kennedy GT, Owen DM, Grant DM, Magee AI, et al (2008) A compact, multidimensional spectrofluorometer exploiting supercontinuum generation. J Biophotonics 1: 494–505. 19343675 10.1002/jbio.200810051

[35] Tsien R, Pozzan T (1989) MEASUREMENT OF CYTOSOLIC FREE CA-2+ WITH QUIN2. Methods in Enzymology 172: 230–262. 2747529 10.1016/s0076-6879(89)72017-6

[36] Clayton AHA, Hanley QS, Verveer PJ (2004) Graphical representation and multicomponent analysis of single-frequency fluorescence lifetime imaging microscopy data. Journal of Microscopy-Oxford 213: 1–5. 10.1111/j.1365-2818.2004.01265.x 14678506

[37] Verveer PJ, Bastiaens PIH (2003) Evaluation of global analysis algorithms for single frequency fluorescence lifetime imaging microscopy data. Journal of Microscopy-Oxford 209: 1–7. 10.1046/j.1365-2818.2003.01093.x 12535178

[38] Kumar S, Alibhai D, Margineanu A, Laine R, Kennedy G, et al (2011) FLIM FRET Technology for Drug Discovery: Automated Multiwell-Plate High-Content Analysis, Multiplexed Readouts and Application in Situ. Chemphyschem 12: 609–626. 21337485 10.1002/cphc.201000874 PMC3084521

[39] Palmer AE, Tsien RY (2006) Measuring calcium signaling using genetically targetable fluorescent indicators. Nature Protocols 1: 1057–1065. 17406387 10.1038/nprot.2006.172

[40] Swaminathan R, Hoang CP, Verkman AS (1997) Photobleaching recovery and anisotropy decay of green fluorescent protein GFP-S65T in solution and cells: Cytoplasmic viscosity probed by green fluorescent protein translational and rotational diffusion. Biophysical Journal 72: 1900–1907. 9083693 10.1016/S0006-3495(97)78835-0 PMC1184383

[41] Gautier I, Tramier M, Durieux C, Coppey J, Pansu RB, et al (2001) Homo-FRET microscopy in living cells to measure monomer-dimer transition of GFP-tagged proteins. Biophysical Journal 80: 3000–3008. 11371472 10.1016/S0006-3495(01)76265-0 PMC1301483

[42] Subramaniam V, Hanley QS, Clayton AHA, Jovin TM (2003) Photophysics of green and red fluorescent proteins: Implications for quantitative microscopy. Biophotonics, Pt A 360: 178–201. 10.1016/s0076-6879(03)60110-2 12622150

[43] Tramier M, Zahid M, Mevel JC, Masse MJ, Coppey-Moisan M (2006) Sensitivity of CFP/YFP and GFP/mCherry pairs to donor photobleaching on FRET determination by fluorescence lifetime imaging microscopy in living cells. Microscopy Research and Technique 69: 933–939. 16941642 10.1002/jemt.20370

[44] Sun YS, Wallrabe H, Booker CF, Day RN, Periasamy A (2010) Three-Color Spectral FRET Microscopy Localizes Three Interacting Proteins in Living Cells. Biophysical Journal 99: 1274–1283. 20713013 10.1016/j.bpj.2010.06.004 PMC2920763

[45] Sun YS, Booker CF, Kumari S, Day RN, Davidson M, et al (2009) Characterization of an orange acceptor fluorescent protein for sensitized spectral fluorescence resonance energy transfer microscopy using a white-light laser. Journal of Biomedical Optics 14. 10.1117/1.3227036 PMC2774974 19895111

[46] Kim J, Kwon D, Lee J, Pasquier H, Grailhe R (2009) The use of cyan fluorescent protein variants with a distinctive lifetime signature. Molecular Biosystems 5: 151–153. 19156260 10.1039/b815445g

[47] Millington M, Grindlay GJ, Altenbach K, Neely RK, Kolch W, et al (2007) High-precision FLIM-FRET in fixed and living cells reveals heterogeneity in a simple CFP-YFP fusion protein. Biophysical Chemistry 127: 155–164. 17336446 10.1016/j.bpc.2007.01.008

[48] Seifert MH, Ksiazek D, Azim MK, Smialowski P, Budisa N, et al (2002) Slow exchange in the chromophore of a green fluorescent protein variant. Journal of the American Chemical Society 124: 7932–7942. 12095337 10.1021/ja0257725

[49] Koushik SV, Chen H, Thaler C, Puhl HL, Vogel SS (2006) Cerulean, Venus, and Venus(Y67C) FRET reference standards. Biophysical Journal 91: L99–L101. 17040988 10.1529/biophysj.106.096206 PMC1779932

[50] Koushik SV, Blank PS, Vogel SS (2009) Anomalous Surplus Energy Transfer Observed with Multiple FRET Acceptors. Plos One 4. 10.1371/journal.pone.0008031 PMC2778011 19946626

[51] Padilla-Parra S, Auduge N, Lalucque H, Mevel JC, Coppey-Moisan M, et al (2009) Quantitative Comparison of Different Fluorescent Protein Couples for Fast FRET-FLIM Acquisition. Biophysical Journal 97: 2368–2376. 19843469 10.1016/j.bpj.2009.07.044 PMC2764072

