# Supplementary material for: Fluorescence Lifetime Readouts of Troponin-C-Based Calcium FRET Sensors: A Quantitative Comparison of CFP and mTFP1 as Donor Fluorophores
Source: PLoS One. 2012 Nov 9;7(11):e49200. doi: 10.1371/journal.pone.0049200 (PMC3494685; doi:10.1371/journal.pone.0049200)
Supplement: Figure S1 — Sequences of the forward and reverse primers for the creation of mTFP-TnC-Cit from TN-L15 and pmTFP-N. (PDF) [file pone.0049200.s001.pdf]

Forward primer: AGACTGGATCCTCGAATTCG **BamHI** restriction site underlined

Reverse primer: TCTGCATGCCCTTGTACAGC **SphI** restriction site underlined
