# Supplementary figures and images for: Fluorescence Lifetime Readouts of Troponin-C-Based Calcium FRET Sensors: A Quantitative Comparison of CFP and mTFP1 as Donor Fluorophores
Source: PLoS One. 2012 Nov 9;7(11):e49200. doi: 10.1371/journal.pone.0049200 (PMC3494685; doi:10.1371/journal.pone.0049200)

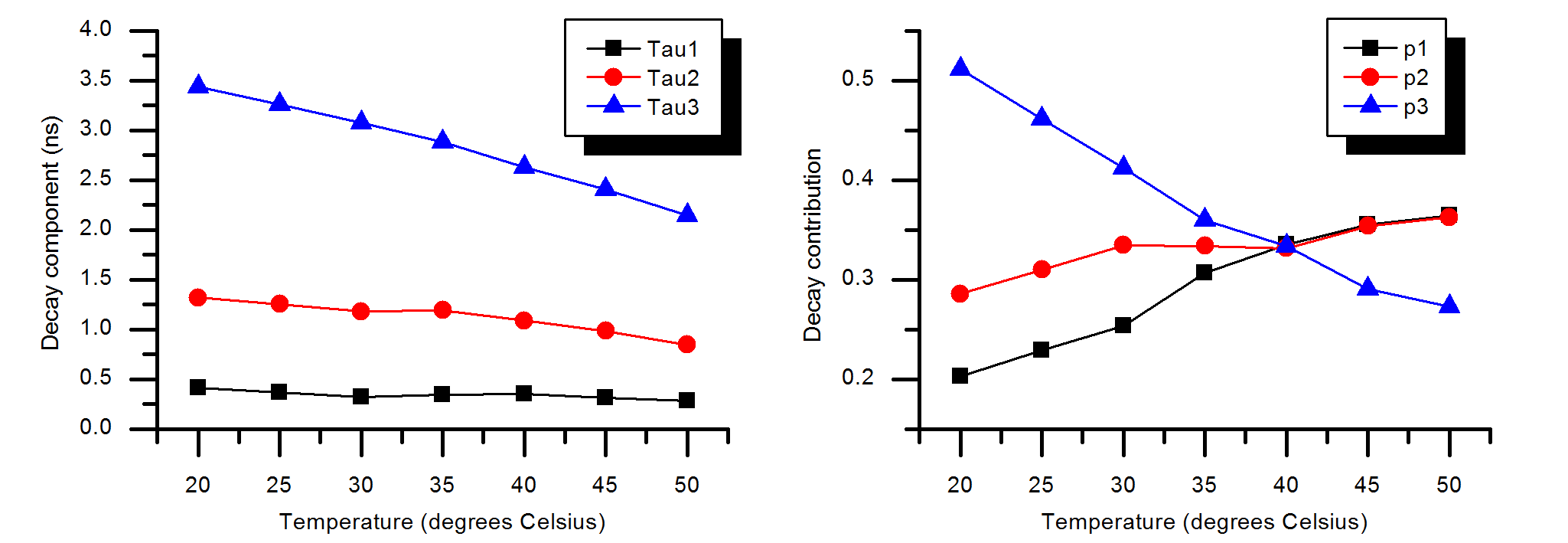

Supplement: Figure S3 — Temperature dependence of ΔC11CFP decay components (left) and contributions (right). All decays were fitted with a triple exponential model. (TIF) [file pone.0049200.s003.tif]

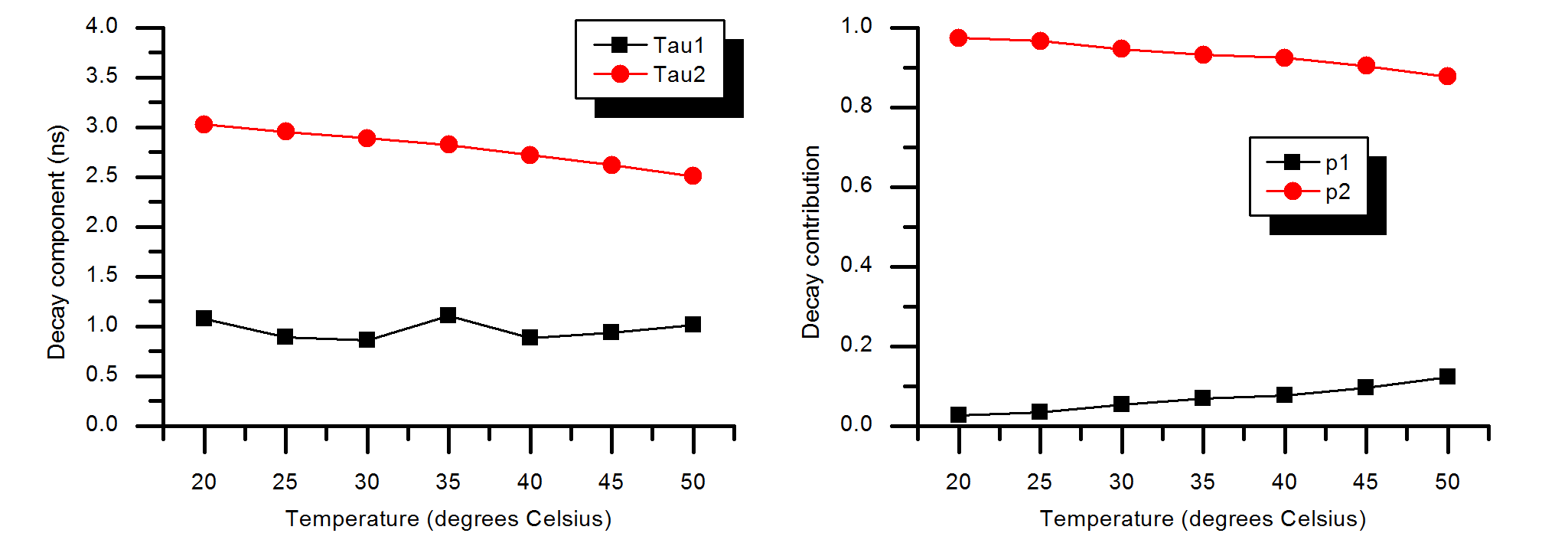

Supplement: Figure S4 — Temperature dependence of mTFP1 decay components (left) and contributions (right). All decays were fitted with a double exponential model. (TIF) [file pone.0049200.s004.tif]

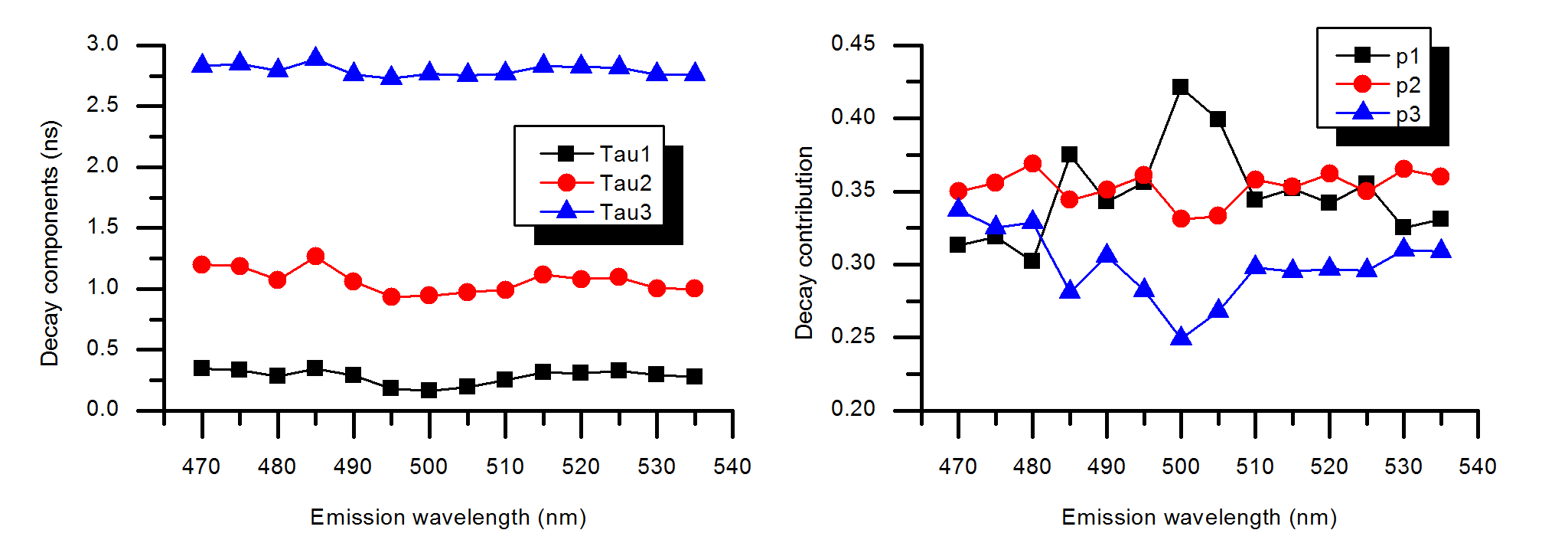

Supplement: Figure S5 — ΔC11CFP decay components (left) and their contributions (right) at different emission wavelength. All decays were fitted with a triple exponential model. (TIF) [file pone.0049200.s005.tif]

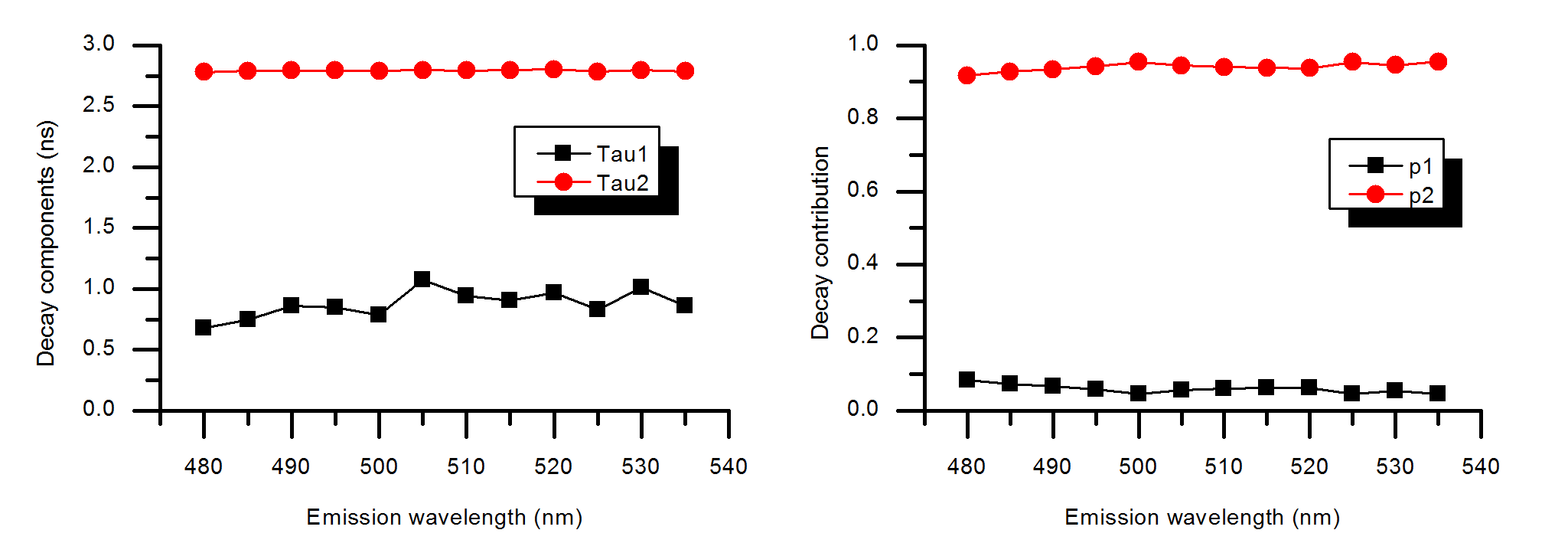

Supplement: Figure S6 — mTFP1 decay components (left) and their contributions (right) at different emission wavelength. All decays were fitted with a double exponential model. (TIF) [file pone.0049200.s006.tif]
